# Photocatalytic Degradation of Bacterial Lipopolysaccharides by Peptide-Coated TiO_2_ Nanoparticles

**DOI:** 10.1021/acsami.4c15706

**Published:** 2024-10-23

**Authors:** Lucrezia Caselli, Guanqun Du, Samantha Micciulla, Tanja Traini, Federica Sebastiani, Ragna Guldsmed Diedrichsen, Sebastian Köhler, Maximilian W. A. Skoda, Mariena J. A. van der Plas, Martin Malmsten

**Affiliations:** † Department of Physical Chemistry 1, Lund University, Lund SE-22100, Sweden; ‡ Institut Laue−Langevin, CS 20156, Grenoble, Cedex 9 38042, France; § Department of Pharmacy, University of Copenhagen, Copenhagen DK-2100, Denmark; ∥ LINXS Institute of Advanced Neutron and X-ray Science, Scheelevagen 19, Lund 22370, Sweden; ⊥ ISIS Pulsed Neutron and Muon Source, Rutherford Appleton Laboratory, Harwell OX11 0QX, U.K.

**Keywords:** antimicrobial peptide, lipopolysaccharide, photocatalysis, oxidation, TiO_2_

## Abstract

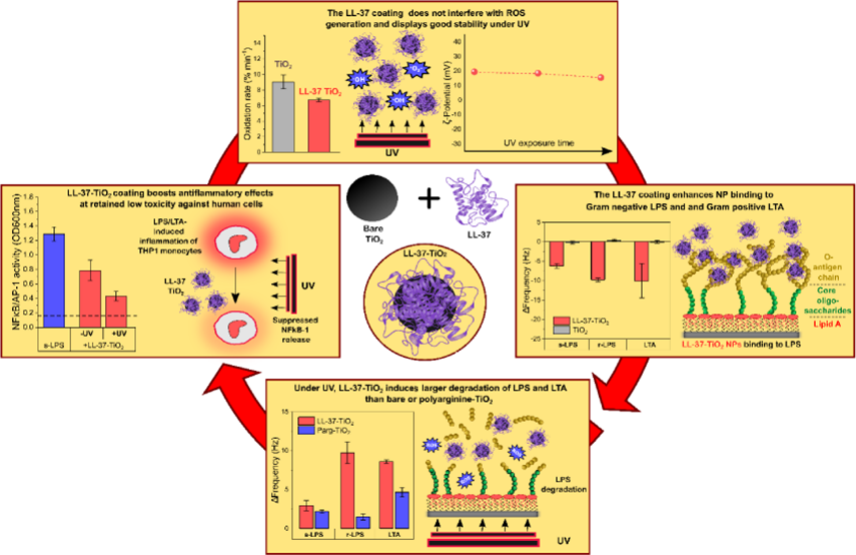

In this study, we report the degradation of smooth and rough lipopolysaccharides (LPS) from Gram-negative bacteria and of lipoteichoic acid (LTA) from Gram-positive bacteria by peptide-coated TiO_2_ nanoparticles (TiO_2_ NPs). While bare TiO_2_ NPs displayed minor binding to both LPS and LTA, coating TiO_2_ NPs with the antimicrobial peptide LL-37 dramatically increased the level of binding to both LPS and LTA, decorating these uniformly. Importantly, peptide coating did not suppress reactive oxygen species generation of TiO_2_ NPs; hence, UV illumination triggered pronounced degradation of LPS and LTA by peptide-coated TiO_2_ NPs. Structural consequences of oxidative degradation were examined by neutron reflectometry for smooth LPS, showing that degradation occurred preferentially in its outer O-antigen tails. Furthermore, cryo-TEM and light scattering showed lipopolysaccharide fragments resulting from degradation to be captured by the NP/lipopolysaccharide coaggregates. The capacity of LL-37-TiO_2_ NPs to capture and degrade LPS and LTA was demonstrated to be of importance for their ability to suppress lipopolysaccharide-induced activation in human monocytes at simultaneously low toxicity. Together, these results suggest that peptide-coated photocatalytic NPs offer opportunities for the confinement of infection and inflammation.

## Introduction

As a result of antibiotic resistance development, bacterial infections are on their way to once again becoming a leading cause of severe illness and death, even in developed countries.^1^ It is, therefore, urgent to develop novel antimicrobial therapeutics. Besides pharmaceuticals, nanomaterials may display potent antimicrobial properties, also against antibiotic-resistant strains.^1,2^ Among nanomaterials, those displaying photocatalytic activity currently attract considerable interest. In photocatalysis, light triggers the formation of excited electrons and holes, which may react with water, dissolved oxygen, or solutes to form reactive oxygen species (ROSs) at the nanoparticle surface. ROSs are highly reactive and may degrade bacterial phospholipids, lipopolysaccharides, proteins, or DNA.^3^ As a result, photocatalytic nanoparticles (NPs) may display potent antimicrobial effects.^4^ However, the mechanistic understanding of such effects remains elusive.

Compared to human cells, bacterial membranes are dominated by anionic phospholipids and do not contain any sterols.^5−7^ In addition, bacterial membranes are rich in bacterial lipopolysaccharides. Thus, LPS and lipoteichoic acid (LTA) are abundant in the membranes of Gram-positive bacteria, respectively.^8,9^ As LTA also belongs to the family of lipopolysaccharides, both LPS and LTA are generically referred to as “lipopolysaccharides” throughout the manuscript. Due to their amphiphilicity and net negative charge, lipopolysaccharides interact strongly with cationic and amphiphilic compounds, such as antimicrobial peptides (AMPs).^10,11^ In fact, AMP binding to bacterial lipopolysaccharides plays a key role in the mode-of-action of such peptides.^12,13^ Considering the pronounced lipopolysaccharide binding of AMPs, coating photocatalytic NPs with AMPs may thus represent an interesting approach for effectively targeting such nanomaterials toward bacterial membranes.

Recently, we reported on coating photocatalytic NPs with AMPs for the selective oxidative degradation of bacteria-like membranes. In doing so, we found that peptide coating enhanced binding to bacteria-like phospholipid bilayers and caused selective degradation of these, which boosted the antimicrobial effects while simultaneously exerting low cell toxicity.^14^ Investigating how such effects depend on the properties of peptides used for nanoparticle coating, we very recently compared the effects of the AMP KYE21 and its hydrophobized WWWKYE21 variant, showing not only that (i) peptide-coated TiO_2_ nanoparticles displayed similar selectivity between bacteria and human cells as the free peptides and (ii) binding of WWWKYE21–TiO_2_ to bacteria-like membranes was higher than that of KYE21-TiO_2_, as were antimicrobial effects, but also that (iii) photocatalytic saturation occurs at very high nanoparticle binding density due to light scattering.^15^

Building on this previous work, further work is needed since very little is known about the effects of the lipopolysaccharide structure on its degradation by photocatalytic NPs and about the consequences of lipopolysaccharide degradation for cell activation and inflammation.^11^ Thus, while complete degradation of lipopolysaccharides can be expected to result in efficient suppression of cell activation, lipopolysaccharide fragments released during the degradation process may still be inflammatory. Incomplete lipopolysaccharide degradation and/or residue capture may therefore aggravate the situation, analogous to inflammation triggered by *Klebsiella pnumoniae* bacteria on incomplete antibiotic exposure.^16^ Addressing this knowledge gap, we set out to address the following research questions: (i) how does oxidative degradation of bacterial lipopolysaccharides depend on their structure, (ii) can oxidative degradation of bacterial lipopolysaccharides be boosted by AMP coating of photocatalytic NPs, and (iii) how does this translate into anti-inflammatory effects and cell toxicity?

In doing so, we focus on TiO_2_ NPs, for which potent antimicrobial effects have been previously reported.^4^ The large band gap of TiO_2_^17^ necessitates UV exposure for photocatalytic effects. While this may be a disadvantage from an application perspective, it allows particle–membrane interactions “in darkness” to be conveniently differentiated from oxidation effects, which is important for dissecting mechanistic aspects. As an antimicrobial peptide, LL-37 (LLGDFFRKSKEKIGKEFKRIVQRIKDFLRNLVPRTES) was chosen, as this is a widely used benchmark peptide.^18^ A reason for this is that LL-37 displays potent antimicrobial effects as well as a range of other host defense effects, which may be refined or boosted, e.g., by truncations, amino acid substitutions, or chemical modifications.^19,20^ This peptide was also used in our previous study of oxidative degradation of bacterial lipid membranes by AMP-coated TiO_2_ NPs,^14^ thus enabling the comparison of results for two key components in bacterial cell walls, i.e., phospholipids and lipopolysaccharides. Complementing this, studies were performed for the polyarginine homopolypeptide, allowing the effects of positive charges alone for the effects above to be elucidated. Regarding lipopolysaccharides, studies were performed for both “rough” and “smooth” forms of Gram-negative LPS (“smooth” characterized by longer polysaccharide moieties and a lower negative charge density than “rough”),^21^ as well as for Gram-positive LTA (having a smaller hydrophobic acyl moiety than Gram-negative LPS.^22^

For these systems, we employed a battery of physicochemical approaches to monitor ROS generation, nanoparticle binding to lipopolysaccharides, and resulting coaggregate formation, as well as structural aspects of nanoparticle binding and lipopolysaccharide degradation, all in the absence and presence of NP peptide coating as well as UV illumination. For monitoring how such physicochemical properties translate into anti-inflammatory effects of bare and peptide-coated TiO_2_ nanoparticles, LPS/LTA-induced cell activation and toxicity were investigated for human monocytes. Considering the systemic nature of inflammation, the study was performed at physiologic pH 7.4.^23−25^

## Materials and Methods

### Materials

Anatase TiO_2_ NPs (4–8 nm) were supplied by a PlasmaChem GmbH. TiO_2_ NPs were previously characterized by cryo-TEM and small-angle X-ray scattering (SAXS).^14^ LL-37 (LLGDFFRKSKEKIGKEFKRIVQRIKDFLRNLVPRTES; >95%) was from Thermo Fisher, as was C_11_-BODIPY 581/591. Polyarginine (Mw 5–15 kDa), octadecyltrichlorosilane (OTS), smooth LPS from *Escherichia coli* (*E. coli*) O111:B4, rough LPS from *E. coli* F583, lipid A from *E. coli* F583, and LTA from *Staphylococcus aureus* (*S. aureus*) were all purchased from Sigma-Aldrich. Phospholipids were purchased from Avanti Polar Lipids. Ultrapure Milli-Q water (MQ, 18.2 MΩ cm) and D_2_O (99% deuterated, Sigma-Aldrich) were used throughout.

### Liposome Preparation

Liposomes for ROS studies were prepared as described previously.^16^ Briefly, 10 mg/mL lipid stocks in chloroform were mixed with 50/25/25 (mol/mol) POPC/PAPC/POPG (+PG). Stock samples were dried under N_2_ and kept under vacuum for 2 h. The films thus formed were rehydrated in 10 mM Tris, pH 7.4 (referred to as “Tris” below), to 1 mg/mL. The dispersion was bath-sonicated and then extruded through polycarbonate filters (Ø 100 nm, LipoFast miniextruder (Avestin)).

### Preparation of NP-LPS and NP-LTA Mixtures

Mixed NP-LPS or NP-LTA samples were prepared 1:1 (wt) by dropwise addition of 25 μL of LPS or LTA (4000 ppm (4 mg/mL) in Tris) to 975 μL of NPs (100 ppm (0.1 mg/mL) in Tris), followed by vigorous vortexing. Samples were subsequently used for cryoTEM, SAXS, DLS, and ζ-potential studies.

### Size and ζ-Potential Measurements

Dynamic and electrophoretic light scattering (DLS and ELS; ϕ = 173°) were performed using a Zetasizer Nano ZSP (Malvern Pananalytical Ltd.) to obtain average particle sizes and ζ-potentials. Measurements were taken at 25 °C.

### C_11_-BODIPY 581/591 Oxidation Assay

Oxidation assay was performed as outlined before.^26,27^ In short, C_11_-BODIPY 581/591 was incorporated into +PG vesicles, which were then exposed to UV illumination (Spectroline ENF-260C, 254 nm; 3 mW/cm^2^) with or without TiO_2_ NPs (100 ppm). Fluorescence spectra (λ_ex_ = 485 nm) were obtained using a Cary Eclipse spectrophotometer (Agilent Technologies), and oxidation was quantified by red (λ_max_ = 594 nm) to green (λ_max_ = 520 nm) emission in the presence and absence of ROS scavengers.^28,29^ Measurements were performed at 37 °C.

### Cryogenic Transmission Electron Microscopy

Cryo-TEM experiments were performed by using a JEM-2200FS microscope (JEOL). A TemCam-F416 camera (TVIPS) was employed to record zero-loss images at 200 kV. 4 μL droplets of the samples were deposited on a Lacey Formvar grid (Ted Pella) and blotted with filter paper. The grid was then plunged into liquid ethane (−180 °C) for vitrification, stored in liquid nitrogen (−196 °C), and transferred to a microscope (Fischione Model 2550) right before image acquisition. Image analysis was performed using ImageJ.^30^

### Synchrotron Small-Angle X-Ray Scattering

Structural features of lipopolysaccharide-NP coaggregates were studied through Synchrotron SAXS experiments at the BioSAXS beamline BM29 (ESRF, Grenoble, France).^31^ An X-ray monochromatic radiation (12.46 keV) was employed to collect the scattering profiles in the 0.05 ≤ *q* ≤ 5 nm^–1^ range with a Pilatus3 2M detector operating in vacuum at 2.849 m. Bare TiO_2_ NPs and mixed NP-LPS or NP-LTA samples (before and after UV illumination) were pipetted from Eppendorf PCR tube strips by an automatic sample changer and transferred in a quartz glass capillary (Ø 1 mm) for scattering acquisition. Thirty 2D images (0.5 s exposure) were collected for each sample and for the buffer at 25 °C. Data were automatically processed to provide buffer-subtracted averaged scattering profiles.^32^

### Quartz Crystal Microbalance with Dissipation Monitoring

QCM-d measurements were performed as described previously^26,27^ using a QSense Analyzer (Biolin Scientific). LPS and LTA layers were formed on hydrophobic polystyrene-coated surfaces (QSense QSX 305 PS-Hydrophobic Polystyrene). The contact angle of water at such polystyrene surface was ∼92° at room temperature, as previously determined.^33^ Cells and tubings were cleaned with 2% Hellmanex^15^ and dried under N_2_. Polystyrene-coated surfaces were then mounted into the measurement chambers. Dispersions of smooth LPS, lipid A, and LTA were prepared in Tris, 150 mM NaCl, at 400 ppm, while a higher concentration (1000 ppm) was needed for rough LPS to form a dense monolayer. Triethylamine (4% w/w) was added to the lipid A dispersion to ensure solubility in the aqueous buffer.^34^ Samples were tip-sonicated for 5 min^15^ and subsequently injected using a peristaltic pump. A liquid flow rate of 0.1 mL/min was employed throughout the experiments. Sample deposition and layer formation were monitored from frequency shifts (Δ*F*) and dissipation changes (Δ*D*). This was followed by rinsing with Tris and 150 mM NaCl to remove excess sample. Subsequently, bare or peptide-coated TiO_2_ NPs (100 ppm) were flushed into the measurement chamber, followed by exposure to UV light for 2 h (Spectroline lamp ENF-260C, 254 nm, 3 mW/cm^2^).

### Neutron Reflectometry

The structure of supported LPS layers was characterized by NR, employing the D17 reflectometer (Institut Laue-Langevin).^35^ The *Q*-region of interest (∼0.01 to 0.3 Å^–1^) was covered using incident angles of 0.8° and 3.0°. NR experiments on smooth LPS interacting with TiO_2_ NPs were performed on the OffSpec reflectometer (ISIS Pulsed Neutron and Muon Source^36^). For these experiments, incident angles of 0.3°, 1.0°, and 2.3° were used to cover the *Q*-region (≈0.01 to 0.35 Å^–1^). Flow cells, the top plate of which had a Ø 30 mm opening, were used in combination with UV-transparent quartz blocks (RMS < 4.5 Å, PI-KEM Ltd.) for in situ UV irradiation. The blocks were cleaned as described previously.^23^ After that, they were dried at 100 °C and transferred to 1 mM OTS in toluene, followed by incubation (1 h) in a glovebox under N_2_ flow.^37,38^ This procedure allowed the formation of a hydrophobic OTS monolayer onto the block surface, characterized by the ∼100° water contact angle.^39^ HPLC tubing, PEEK troughs, and O-rings were cleaned with 2% Hellmanex (Hellma Analytics).^15^

OTS-coated surfaces were characterized in three contrasts, i.e., Tris, 150 mM NaCl in MQ (h-Tris), D_2_O (d-Tris), and 68.6/31.4% v/v D_2_O/MQ, to match the scattering length density (SLD) of the quartz substrate (qm-Tris). The cells were then rinsed, after which smooth LPS (1000 ppm in h-Tris) was manually injected as described previously. The layers thus formed were characterized as h-, qm-, and d-Tris. After that, 15 mL of bare or LL-37-coated TiO_2_ NPs (100 ppm in h-Tris) were injected manually, followed by incubation (10 min) and flushing by h-Tris to remove excess NPs, after which samples were characterized in h-, qm-, and d-Tris. The systems were then subjected to in situ UV irradiation (Spectroline Lamp ENF-260C, 254 nm, 3 mW/cm^2^) for 2 h. Immediately after UV exposure, samples were rinsed with 20 mL of d-Tris (1 mL/min) and measured in three contrasts. Experimental NR profiles were fitted by Motofit within IGOR Pro.^40^ The best fits were converted to SLD profiles perpendicular to the surface. To minimize fitting uncertainty, data were refitted 200 times by Monte Carlo error analysis.^41^

### Cell Experiments

Human THP1 monocytes (InvivoGen) were employed to measure the NP effects of lipopolysaccharide-induced NF-κB activation. To this purpose, 100 ppm of NPs were mixed with 100 ppm-smooth LPS or rough LPS or 5000 ppm LTA in Tris in quartz cuvettes. Samples were then incubated for 2 h at room temperature with or without UV illumination (Spectroline ENF-260C, 254 nm, 3 mW/cm^2^). Cells (1 × 10^6^ cells/mL) were incubated with these samples after dilution in Tris to 0.1 ppm for LPS or 5 ppm for LTA. After incubation in 5% CO_2_ at 37 °C for 18–20 h, NF-κB activation was determined by the detection substrate (Quanti-Blue, InvivoGen) at 600 nm. To monitor cell toxicity, a lactic acid dehydrogenase (LDH) assay was employed. For this, THP1 cells were incubated as above, and LDH release was measured using a lactate dehydrogenase assay kit (Invitrogen CyQUANT LDH Cytotoxicity Assay (Thermo Fisher).

### Statistical Analysis

If not stated otherwise, data are reported as means ± standard error of mean (SEM). For all experiments (except for NR and cryo-TEM experiments), measurements were conducted in at least triplicate. For the NR, errors associated with the structural parameters were obtained as described above. For cryo-TEM, a minimum number of 20 images were acquired for each sample to achieve statistically significant morphological and structural information.

## Results and Discussion

### Peptide Coating of TiO_2_ NPs

**Figure 1:**
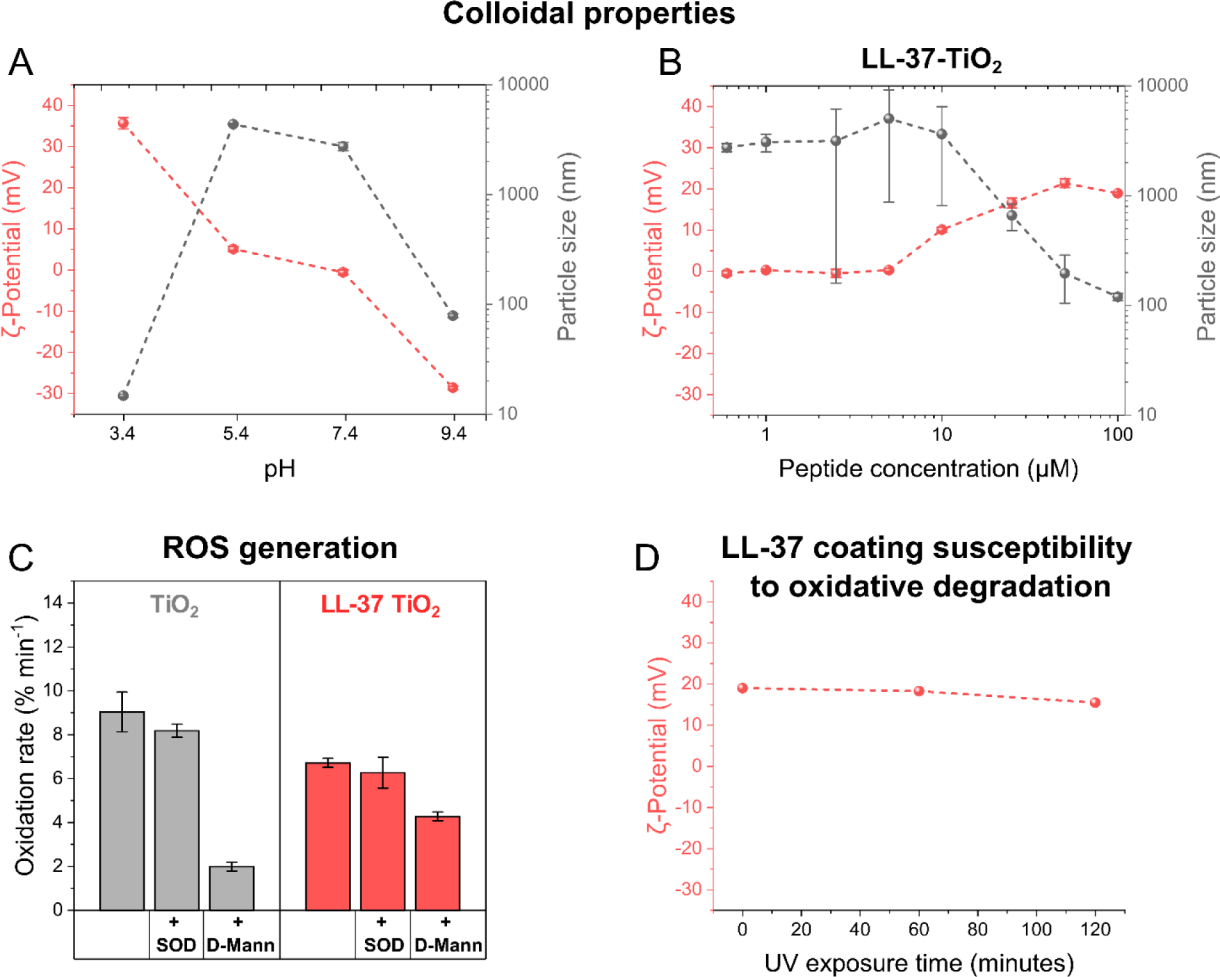
Characterization of LL-37-TiO_2_ NPs. (A) ζ-potential and average particle size of bare TiO_2_ NPs (100 ppm) in 10 mM acetate (pH 3.4 and 5.4) or 10 mM Tris (pH 7.4 and 9.4). (B) ζ-potential and average particle size of TiO_2_ nanoparticles (100 ppm) loaded at varying concentrations of LL-37 in 10 mM Tris, pH 7.4 (*n* = 3). (C) C_11_–BODIPY oxidation rates for bare or LL-37-coated TiO_2_ NPs for +PG LUVs subjected to in situ UV exposure in 10 mM Tris, pH 7.4 (*n* = 3). Corresponding oxidation kinetics are shown in Figure S1. (D) ζ-potential of TiO_2_ NPs coated with 50 μM LL-37 in 10 mM Tris, pH 7.4, before and after 1 or 2 h of UV illumination. Results are means ± SEM of *n* = 3 experiments.

TiO_2_ NPs have an isoelectric point (IEP) of pH 6–6.5.^42^ Mirroring this, the TiO_2_ NPs employed in the present study displayed a positive ζ-potential at pH 3.4 and 5.4, a weak negative potential at pH 7.4, and a strongly negative potential at pH 9.4 (Figure 1A). Reflecting this, TiO_2_ NPs underwent aggregation at pH 5.4 and 7.4, but less so at pH 3.4 and 9.4. On coating TiO_2_ nanoparticles with cationic LL-37 at pH 7.4, a concentration-dependent increase in positive ζ-potential was observed (Figure 1B). At saturation binding, a net positive ζ-potential of +22 ± 3 mV was observed for TiO_2_ NPs coated with LL-37. As a result of this, colloidal stability increased significantly at high peptide loading, as seen from the decrease in the effective particle size. Based on these results, full peptide loading was inferred to occur at a peptide concentration of 50 μM, beyond which only marginal effects on the ζ-potential and particle size were observed. Below, TiO_2_ NPs loaded at this peptide concentration are referred to as LL-37-TiO_2_.

Next, we investigated whether peptide loading influenced ROS generation. Since excited electrons and holes need to reach the ambient aqueous solution for ROS formation, it is essential for the photocatalytic effects that the peptide coating allows such reactions to occur. As shown in Figure 1C, LL-37 coating did not markedly suppress ROS generation (see Figure S1A for kinetics). Furthermore, quenching results showed both ^•^OH and ^•^O_2_^–^ to be formed on UV illumination for bare and coated NPs alike (Figure 1C; see Figure S1B,C for kinetics). As ^•^OH and ^•^O_2_^–^ are highly reactive radicals, which are able to degrade proteins and polypeptides through oxidation,^43^ there is a risk that the LL-37 coating formed on TiO_2_ NPs degrades upon UV illumination. Indeed, a minor decrease was observed in the ζ-potential under UV illumination, indicating some peptide degradation and/or oxidation. Importantly, however, LL-37-coated TiO_2_ NPs retained their positive charge also after UV exposure (Figure 1D). Thus, irrespective of degradation, most of the peptide (including any fragments formed during UV illumination) remained bound at the nanoparticle surface, where they provided an electrostatic driving force for binding to negatively charged lipopolysaccharides.

### Nanoparticle Interactions with Gram-Negative LPS and Gram-Positive LTA in Solution

Next, we investigated coaggregates formed by bare or peptide-coated TiO_2_ NPs with anionic LPS or LTA (see Figure S2 for the hydrodynamic size and ζ-potential of LPS and LTA in the absence of NPs). Mixing 100 ppm of LL-37-TiO_2_ with 100 ppm smooth LPS resulted in very large (≈1000 nm) coaggregates of near-neutral surface charge. Similar-sized coaggregates of larger negative surface charge were observed for rough LPS and LTA (Figure S3). A similar behavior was found also for LPS and LTA mixed with bare TiO_2_ NPs, although aggregation by care TiO_2_ NPs alone at this pH obscures effects of lipopolysaccharide-NP coaggregation (Figure 1A). No major variations in aggregate size and ζ-potential were observed after 2 h of UV illumination for any of the samples.

**Figure 2:**
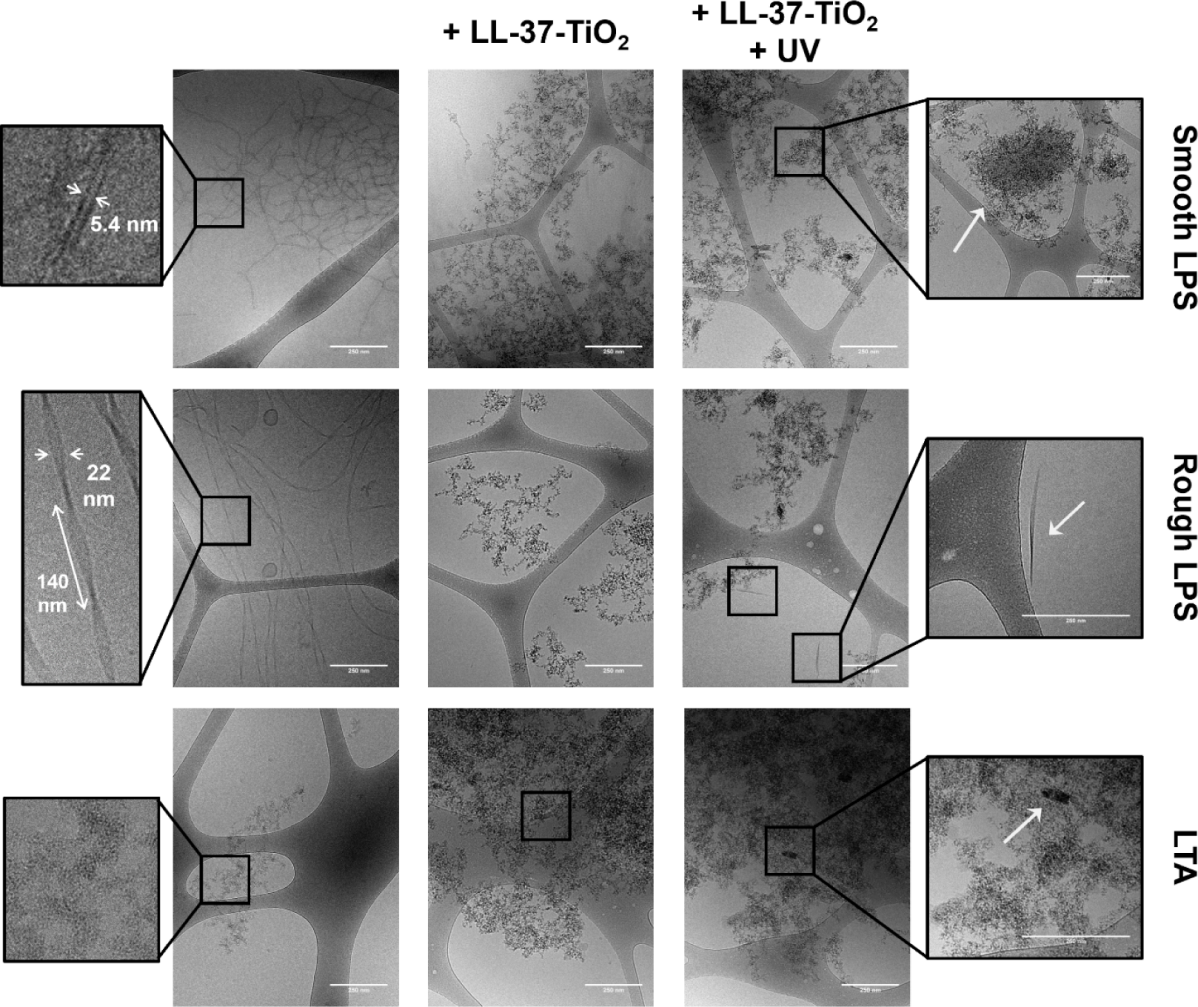
Cryo-TEM images of 100 ppm smooth LPS (top), rough LPS (middle), and LTA (bottom) in 10 mM Tris, pH 7.4 (left), as well as the corresponding systems in the presence of 100 ppm of LL-37-TiO_2_ NPs, either before (middle) and after (right) 2 h of UV illumination. The structural features of LPS and LTA (without LL-37-TiO_2_ NPs) are highlighted in far-left insets, showing the formation of fibrillae with a diameter of ∼5 nm for smooth LPS, twisted ribbon-like structures of ∼22 nm in thickness and ∼140 nm characteristic node-to-node distance for rough LPS, and amorphous nanosized assemblies for LTA. Incubation with LL-37-TiO_2_ NPs leads to aggregates of smooth LPS, rough LPS, and LTA, homogeneously decorated with NPs. UV illumination results in an increased packing density of such aggregates (Figure 3) and triggers the formation of characteristic structures (highlighted in the far-right insets), consisting of amorphous NP-decorated spherical assemblies (mostly observed in smooth LPS samples), free LPS fragments (mostly clearly seen in rough LPS samples), and multilamellar plate-like structures (present in both LPS and LTA samples).

**Figure 3:**
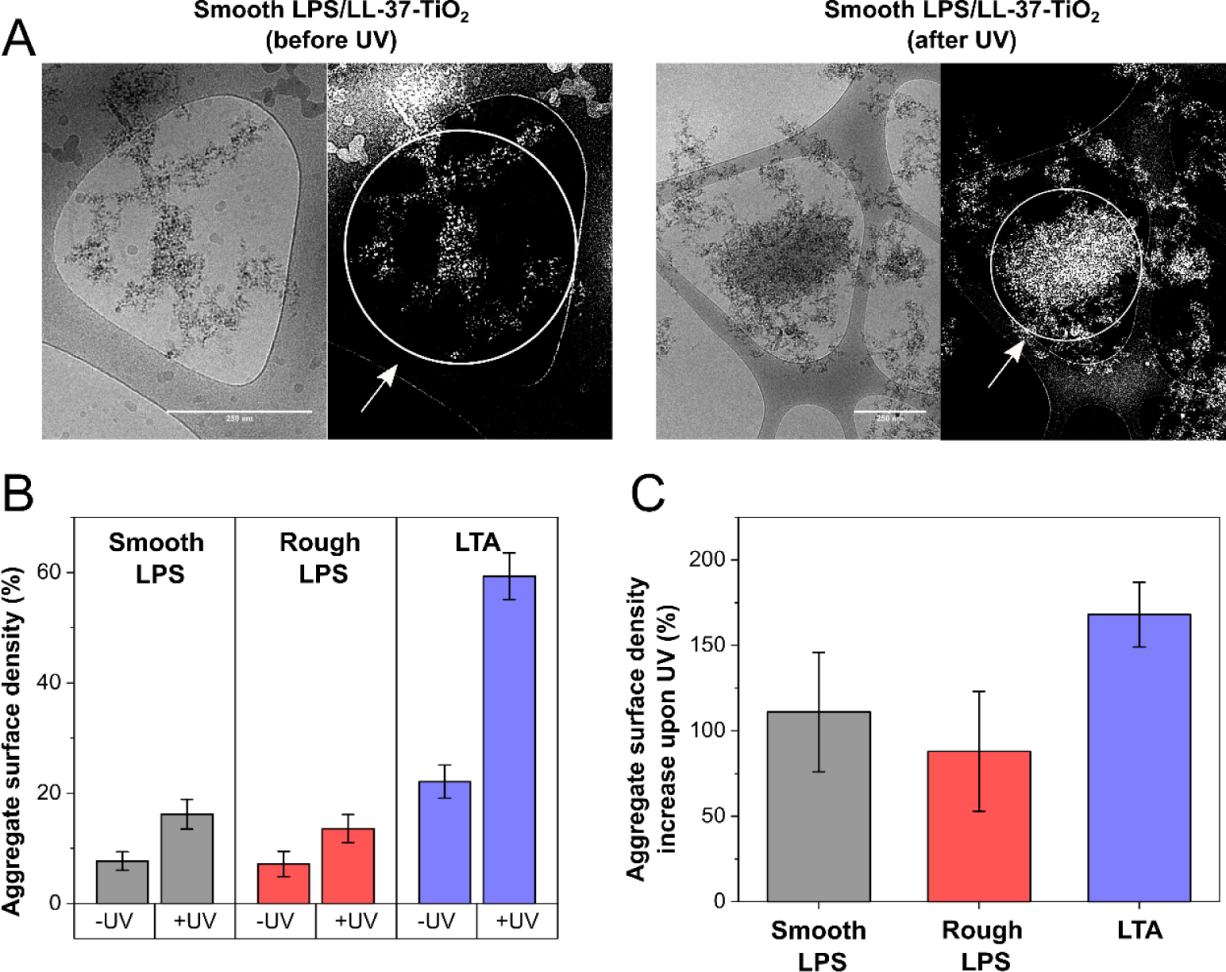
Quantification of the compactness of LL-37-TiO_2_ coaggregates with LPS and LTA obtained through cryo-TEM image analysis with the software ImageJ.^38,39^ The procedure adopted for image analysis is shown in panel A for the representative images of smooth LPS/LL-37-TiO_2_ coaggregates, acquired before (left image) or after (right image) 2 h of UV illumination. Black and white mask images were obtained from original cryo-TEM images to allow precise mapping of the edges of LL-37-TiO_2_/LPS or -LTA coaggregates over the cryo-TEM grid. LL-37-TiO_2_/LPS or -LTA aggregates were analyzed individually by selecting specific portions of the mask (e.g., areas highlighted in white in panel A). For aggregates smaller than the typical size of the holes in the cryo-TEM grid, the smallest circular areas fully enclosing the edges of single aggregates were selected. For aggregates exceeding this size, the largest circular areas enclosed within single grid holes were analyzed. The surface density of the aggregates (%) was then obtained over such selected portions as the ratio between the area occupied by the aggregate (in white) and the empty area (in black) and averaged over a large number of aggregates. Aggregate surface densities (%) for the different samples, before and after UV illumination, are reported in panel B. Shown in panel C are also the percentage increases in the aggregates surface density (%) on UV exposure. Results reported in panels B and C are means ± SEM of *n* ≥ 20 aggregates per sample.

To elucidate the structure of coaggregates formed by LPS or LTA with peptide-coated TiO_2_ NPs, we investigated these by cryoTEM. As shown in Figure 2, both smooth and rough LPS, as well as LTA, form thread-like structures in line with previous reports.^44−46^ Note, however, that lipopolysaccharide aggregates are not equilibrium structures; thus, their self-assembly structure depends on the preparation method. For example, smaller and less thread-like structures can be obtained after repeated extrusion or tip sonication.^47−49^ On addition of LL-37-TiO_2_ NPs (Figure 2), the latter distributed homogeneously over the fiber structures of both smooth and rough LPS, as well as LTA, and triggered the formation of dense coaggregates. Furthermore, scattered multilamellar structures were observed in the presence of LL-37-TiO_2_, as reported previously in the presence of free peptide.^50^ On UV illumination, the coaggregates packed more densely (Figure 3), likely as a result of degradation of some of the LPS chains, relaxing the kinetic constraints for dense packing. In line with this, free LPS fragments were observed after illumination, and most clearly seen outside coaggregates formed between LL-37-TiO_2_ NPs and rough LPS (Figure 2). These findings are in line with the dynamic light scattering results discussed above. To more conclusively elucidate if degradation caused the formation of ordered structures, SAXS was employed. However, no ordered structures were observed for either LPS or LTA (Figure S4), possibly due to their long hydrophilic and negatively charged polysaccharide chains^21,22^ and/or due to the signal from these being masked by the broad NP signal.

### Nanoparticle Interactions with Surface-Bound LPS, Lipid A, and LTA

**Figure 4:**
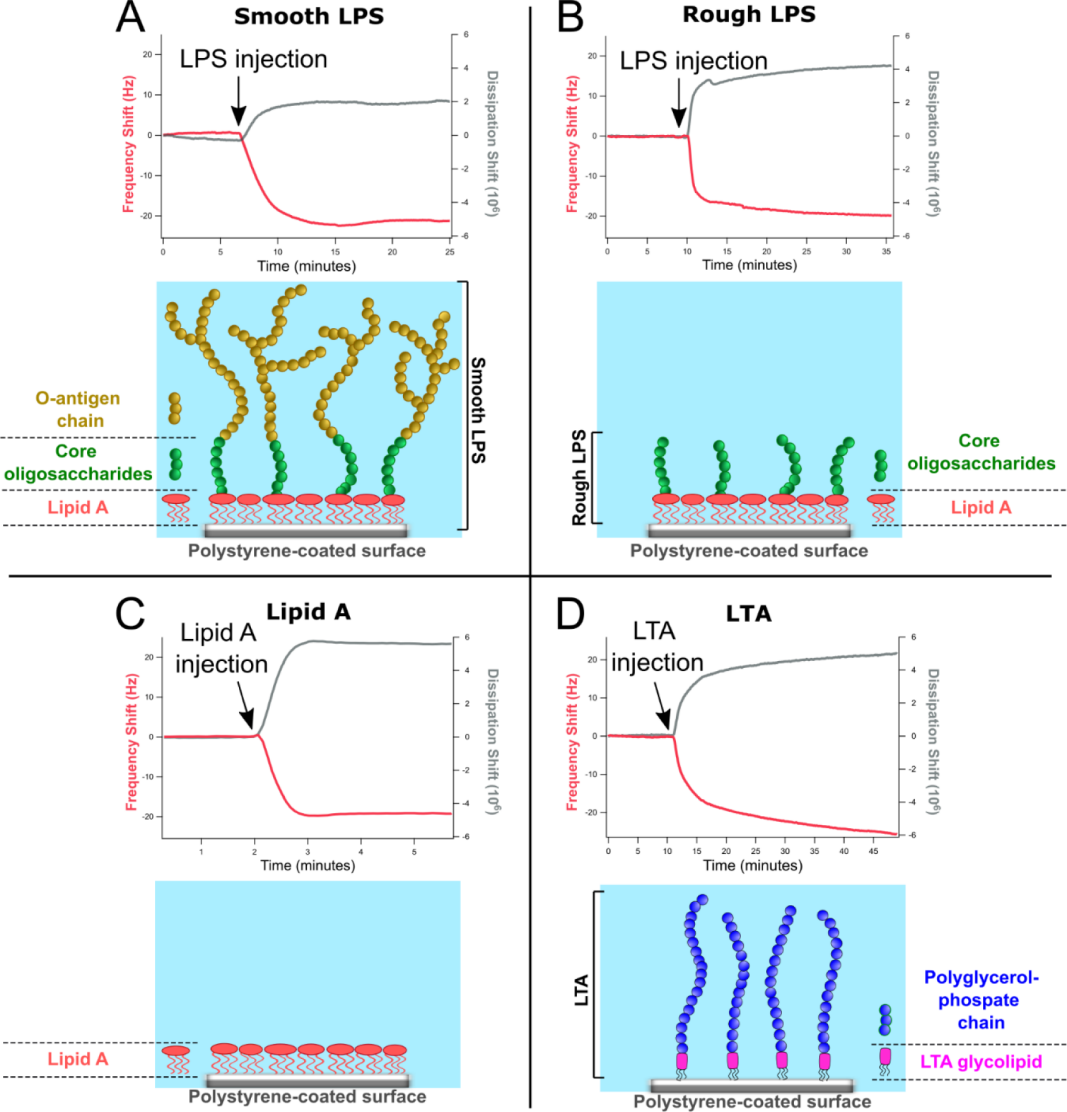
LPS and LTA layer formation. Representative QCM-d kinetics curves showing the 7th overtone of frequency (Δ*F*) and dissipation (Δ*D*) shifts associated with the formation of (A) a smooth LPS layer by deposition on hydrophobic polystyrene from 400 ppm in 10 mM Tris, 150 mM NaCl, pH 7.4; (B) a rough LPS layer on hydrophobic polystyrene from 1000 ppm in 10 mM Tris, 150 mM NaCl, pH 7.4; (C) a lipid A layer on hydrophobic polystyrene by deposition from 400 ppm in 10 mM Tris, 150 mM NaCl, and pH 7.4, containing also triethylamine (4% w/w); and (D) an LTA layer by deposition on hydrophobic polystyrene from 400 ppm in 10 mM Tris buffer, 150 mM NaCl, and pH 7.4.

**Figure 5:**
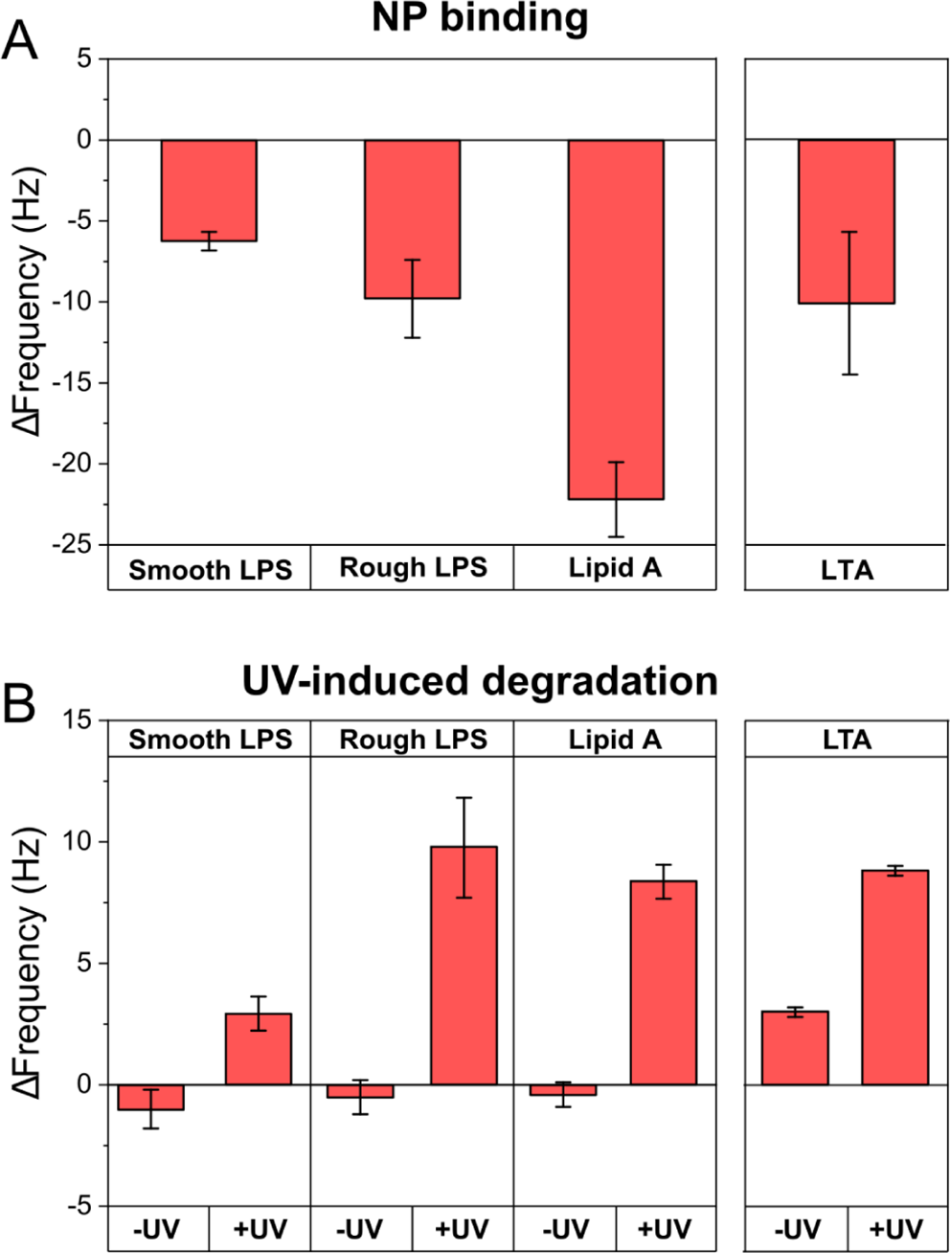
QCM-d results showing frequency shifts caused by (A) binding of 100 ppm LL-37-TiO_2_ NPs to smooth LPS, rough LPS, lipid A, and LTA and (B) effects of 2 h of in situ UV illumination for smooth LPS, rough LPS, lipid A, and LTA interacting with 100 ppm LL-37-TiO_2_. Results are shown for the adsorption to hydrophobic polystyrene surfaces coated with smooth LPS, rough LPS, lipid A, and LTA prepared as in Figure 4. Δ*F* = 0 corresponds to the frequency shift for the smooth LPS, rough LPS, and lipid A layers right before NP addition (A) or UV illumination (B). All measurements were performed in 10 mM Tris + NaCl 150 mM, pH 7.4. Representative QCM-d profiles for panels A and B are shown in Figure S7, while the corresponding results for bare TiO_2_ NPs are shown in Figures S5 and S6. Results are means ± SEM of *n* = 3 experiments.

Mirroring the “end-on” orientation of membrane-bound LPS, we next investigated smooth and rough LPS adsorbed on hydrophobic polystyrene substrates through their hydrophobic lipid A moiety, employing QCM-d. Also, lipid A alone was investigated. While compositions and procedures had to be optimized for the different systems, conditions were found at which all three systems formed well-defined adsorbed layers (Figure 4). On addition of bare TiO_2_ NPs (carrying a weak negative ζ-potential; Figure 1A), very little binding to the negatively charged LPS or lipid A was observed (see Figure S5A for results on binding and Figure S6A for corresponding kinetics; note, however, that NR shows that minor adsorption does in fact occur to these surfaces; cf below). In contrast, LL-37-TiO_2_ NPs displayed pronounced initial binding to both smooth and rough LPS, as well as to lipid A, but then caused partial desorption of rough LPS and lipid A after initial binding (see Figure 5A for results on binding and Figure S7A for the corresponding kinetics). For smooth LPS, no partial desorption was observed, likely due to its longer carbohydrate chains precluding close contact between the peptide-coated nanoparticles and its lipid A moiety (see also the discussion of NR results below).

On UV exposure after LL-37-TiO_2_ NP binding, desorption triggered by oxidative degradation was observed for both smooth and rough LPS, as well as for lipid A (Figures 5B and S7B). Similar results were observed for bare TiO_2_ (see Figure S5B for results on desorption and Figure S6B for the corresponding kinetics). For LL-37-TiO_2_, binding of NP to LTA was comparable with that for rough LPS (Figures 5A and S7A). Also on UV exposure, LTA behaved similarly to LPS (Figures 5B and S7B).

### Structural Aspects of LPS Degradation

**Figure 6:**
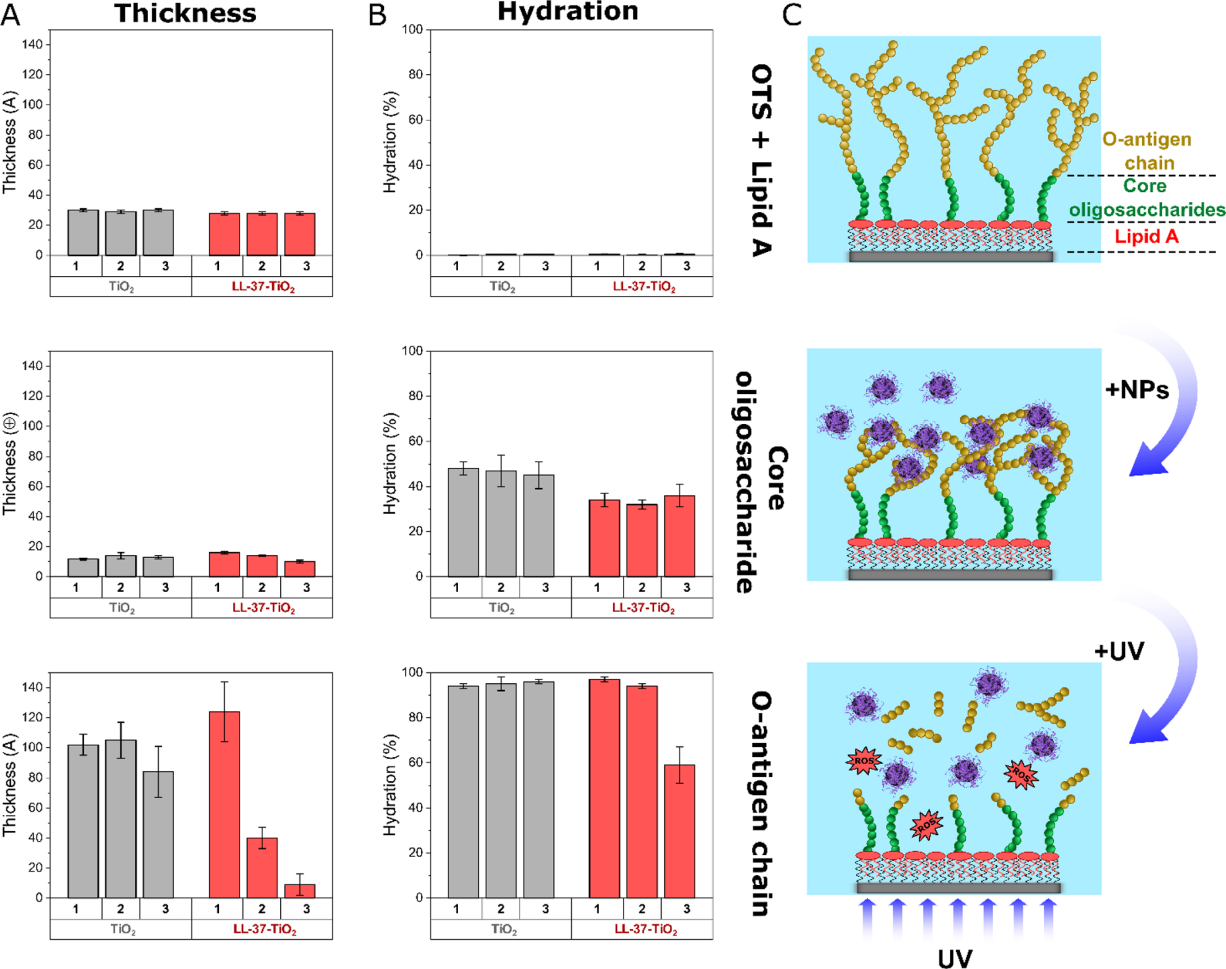
Structural effects on OTS + lipid A (top), core oligosaccharide (middle), and O-antigen chain (bottom) layers induced by bare and LL-37-coated TiO_2_ NPs in the absence and presence of UV illumination. Results were obtained from neutron reflectometry fits, calculating the physical parameters of the bilayers at different time points: (1) before NP incubation, (2) after NP incubation, and (3) after 2 h of in situ UV exposure. Shown are changes in the thickness (A) and hydration (B) for the three different layers, as well as a schematic illustration (C) describing the main structural changes observed for the different LPS domains upon NP interaction and UV illumination. Corresponding experimental curves, best curve fits and calculated SLD profiles, are shown in Figure S9, while Figure S8 collects experimental curves, together with best curve fits and SLD profiles, for the neat OTS layer grafted before LPS deposition.

Next, NR was employed for LL-37-TiO_2_ interacting with smooth LPS. For this, substrates were precoated with a homogeneous hydrophobic layer of OTS^37,49^ as precoating with polystyrene (used in the QCM-d experiments) were incompatible with the high smoothness requirements of NR experiments. The structural parameters of this layer (Figure S8, Table S1) were in agreement with the previous literature.^37,49^ Smooth LPS was then manually injected into the measurement chamber, and the reflectivity changes following the LPS layer formation were monitored. NR profiles (Scheme S1) for smooth LPS interacting with either bare or peptide-coated NPs are shown in Figure S9, together with best curve fits and corresponding SLDs. Key structural data determined through fitting are summarized in Figures 6 and Tables S2 (bare TiO_2_ NPs) and S3 (LL-37-TiO_2_ NPs). Prior to UV illumination, incubation with bare TiO_2_ NPs did not induce significant structural modification in any of the LPS domains. In contrast, the addition of LL-37-TiO_2_ NPs provoked a substantial thickness decrease for the outer O-antigen chains layer, from 124 ± 24 Å to 40 ± 7 Å. UV illumination induced a further decrease (to 9 ± 1 Å) and hydration (from 94 ± 1% to 59 ± 8%), indicating an essentially complete removal of the O-antigen moiety. Contrasting this, only a relatively minor thickness reduction of the O-antigen layer was observed on UV illumination for bare TiO_2_ NPs, from 106 ± 12 Å to 84 ± 17 Å, with negligible hydration changes. For both TiO_2_ and LL-37-TiO_2_ NPs, the layers of the OTS/lipid A and core oligosaccharide were essentially unaffected by UV illumination.

### Anti-Inflammatory Effects and Cell Toxicity

**Figure 7:**
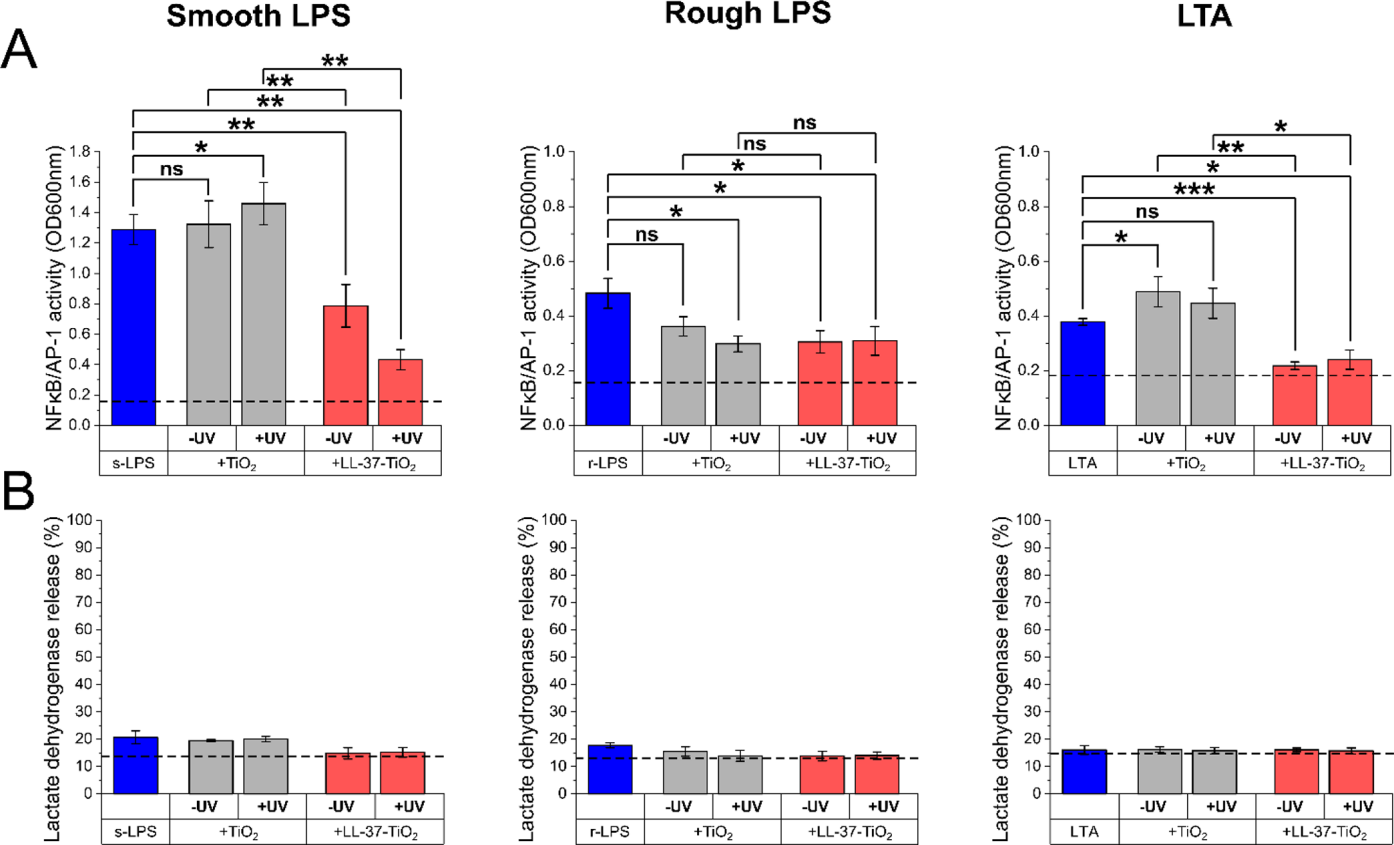
(A) NF-κB/AP-1 activation and (B) LDH release induced by smooth (left) and rough (middle) LPS, as well as LTA (right), in the absence and presence of bare TiO_2_ or LL-37-TiO_2_ NPs in 10 mM Tris, pH 7.4, either before or after 2 h of UV illumination, using THP1-XBlue-CD14 reporter monocytes. The dashed lines in the graphs represent the NF-κB/AP-1 activation or LDH release for the control sample, i.e., 10 mM Tris buffer, pH 7.4, in the absence of LPS, LTA, and NPs. Results are mean ± SEM of 3–7 experiments. Values are significantly different (*p < 0.05, **p < 0.005, and ***p < 0.0005) as analyzed using a one-tailed paired *t* test.

Addressing the biological relevance of the results obtained in the model lipopolysaccharide systems, we next investigated the capacity of the peptide-coated NPs to suppress the activation of human monocytes by LPS and LTA, monitored as a decrease in LPS/LTA-induced NF-kB levels. As shown in Figure 7A, LL-37-TiO_2_ NPs suppressed NF-kB for both smooth and rough LPS, as well as LTA, even without UV illumination. In contrast, minor effects were noted only for rough LPS in the case of bare TiO_2_ NPs. On UV illumination, a further reduction in NF-kB was observed for smooth LPS, whereas bare TiO_2_ NPs did not display any such effect. This is in line with the results obtained for the model systems showing (i) coaggregate formation and resulting lipopolysaccharide confinement for peptide-coated TiO_2_ NPs and (ii) UV-induced lipopolysaccharide degradation. While LL-37-TiO_2_ NPs thus present boosted anti-inflammatory effects compared to bare TiO_2_ NPs, cell toxicity remained low at the level for bare TiO_2_ NPs and lower than that for the negative control (Figure 7B).

## Discussion

Cationic surface modifications of NPs have been widely found to promote their antimicrobial activities.^51^ Such effects are often ascribed to increased binding to negatively charged bacterial membranes. In addition, however, cationic surface modifications of nanoparticles have been frequently reported to display toxicity against human cells.^51,52^ Since conventional surface modifications therefore seem insufficient for antimicrobial effects at simultaneously low toxicity, novel approaches are needed. Surface coating of NPs by AMPs has been suggested as a way to effectively “target” these toward bacteria.^11−14^ Illustrating this, Malekkhaiat-Häffner et al. coated laponite and other nanoclays with LL-37 and found the peptide-coated particles to be potently antimicrobial, yet showing low toxicity against human monocytes.^53^ Conceptually related, Duong et al. reported that coating of polymer particles with GRR10W4 increased uptake into melanoma cells (rich in anionic phospholipids), resulting in enhanced anticancer effects at simultaneously low toxicity against nonmalignant fibroblasts and keratinocytes.^54^

For photocatalytic NPs, AMP coating is inherently more complicated, considering requirements of simultaneous ROS generation and coating robustness against degradation. It is therefore interesting to note that Lu et al.,^55^ Zhang et al.,^56^ and Chen et al.^57^ all reported on advantageous functional performance of peptide-coated photocatalytic nanomaterials as antimicrobials, although not addressing the mechanistic foundation for such effects. In an effort to do so, we recently investigated lipid membrane interactions of TiO_2_-LL-37 NPs by NR in combination with a range of physicochemical methods and biological assays. From this, it was found that (i) AMP coating did not significantly suppress ROS generation and (ii) the peptide coating was quite stable against ROS degradation, allowing (iii) dramatically improved binding to bacteria-like lipid membranes compared to bare TiO_2_ NPs. As a result, (iv) UV-induced membrane degradation was boosted for bacteria-like membranes, whereas human cell-like membranes remained unaffected.^14^ Analogously, LL-37-TiO_2_ NPs were demonstrated antimicrobial effects against both Gram-negative *E. coli* and Gram-positive *S. aureus*, which were substantially boosted on UV illumination. In contrast, LL-37-TiO_2_ NPs caused very little membrane destabilization of human monocytes, demonstrating that antibacterial effects could be reached at simultaneously low cell toxicity.^14^ In the present study, we extend on this previous study and demonstrate that (i) LL-37 coating of TiO_2_ NPs strongly boosts their binding to LPS (lipid A, core oligosaccharide, and O-antigen regions) and LTA alike, (ii) the increased binding of the peptide-coated NPs results in boosted oxidative degradation of all of these, and (iii) fragments formed during photocatalytic degradation are efficiently captured in the lipopolysaccharide-NP coaggregates. As a result of this, the ability of LPS, its different moieties, LTA, and degradation products thereof to activate monocytes is suppressed, as evidenced from suppressed NF-kB generation.

Addressing the effect of peptide properties on photocatalytic antimicrobial effects, we recently performed an investigation of EFK17 (EFKRIVQRIKDFLRNLV), a 17-amino-acid truncation of LL-37. It was found that EFK17 was less efficient than LL-37 in binding to the TiO_2_ NPs, which in turn resulted in lower affinity of the peptide-coated NPs for bacteria-like membranes compared to LL-37, as well as in less efficient photocatalytic degradation and antimicrobial effects. Increasing the hydrophobicity of EFK17 by substituting selected nonionic amino acids with tryptophan residues, however, promoted peptide binding to TiO_2_ NPs, in turn causing higher binding of the peptide-coated NPs to bacterial membranes, as well as boosted antimicrobial effects on UV illumination.^58^ Analogously, we recently investigated membrane interactions of TiO_2_ NPs coated with variants of the peptide KYE21^15^. In doing so, the effects of peptide hydrophobicity were investigated by comparing KYE21 (KYEITTIHNLFRKLTHRLFRR) with a hydrophobically end-tagged variant (WWWKYE21). It was found that increasing the peptide hydrophobicity through the W-tag resulted in increased binding to bacteria-like membranes and membrane components, in turn resulting in boosted photocatalytic degradation, although saturation in photocatalytic effects were observed at high NP concentrations.

In a couple of studies, photocatalytic NPs have been found to suppress inflammation caused by LPS,^59,60^ although the mechanistic origin of such effects remain unresolved. There is also a potential complication of photocatalytic NPs that oxidative degradation may result in the release of bacterial debris, which may potentially trigger inflammation, at least until lipopolysaccharide degradation has progressed sufficiently to result in fragments small enough not to cause cell activation. In Gram-negative bacteria, LPS forms a dense outer barrier, which may act as a barrier for nanoparticles,^61^ preventing proximity between ROS species formed on illumination and the inner plasma membrane. Such effects may contribute to lower antimicrobial effects against Gram-negative reported for photocatalytic NPs.^62^ In relation to this, it is also relevant to consider the relative susceptibility of bacterial membrane components to photocatalytic degradation, i.e., phospholipids, bacterial lipopolysaccharides, and peptidoglycan. Addressing this, Kiwi et al. reported on the photocatalytic oxidation of *E. coli*, as well as of phosphatidylethanolcholine (PE), LPS, and peptidoglycan. The latter was found to be more resistant to degradation than PE and LPS.^63^ Similarly, Liu et al. reported that while photocatalytic action by TiO_2_ NPs was able to damage the outer *E. coli* membrane, it was not able to destroy peptidoglycan, again demonstrating the latter to be more resilient to oxidative disintegration than phospholipids and LPS.^64^

Regarding LPS degradation, Engel et al. found *E. coli* variants with shorter polysaccharide chains to be more susceptible to photocatalytic degradation by carbon nanotubes than those with long ones.^65^ As shown in the present investigation, LL-37-TiO_2_ NPs are able to degrade both the lipid A, the core oligosaccharide, and the O-antigen moieties of LPS. Having said that, NR results show that when all these moieties are present in proper orientation, as for smooth LPS bound to hydrophobic surfaces through its lipid A moiety, LL-37-TiO_2_ NPs preferentially degrade the outer O-antigen moiety. Another key finding of the present investigation is that Gram-positive LTA displays susceptibility to photocatalytic degradation comparable to that of Gram-negative LPS. However, LTA is located within the cross-linked PGN matrix of Gram-negative bacteria. Hence, even if LTA is photocatalytically degraded, it may be kinetically hindered from diffusing out of this matrix, particularly if the peptidoglycan matrix is resilient to photocatalytic degradation. As demonstrated by the findings of this investigation, such kinetic arrest may be promoted by highly cationic peptide coatings.

LTA and LPS both coaggregate strongly with LL-37-TiO_2_ NPs. As such, these lipopolysaccharides are efficiently confined in coaggregates, with very few lipopolysaccharide molecules in the solution surrounding the coaggregates. As a result, lipopolysaccharide-mediated cell activation was suppressed in the presence of LL-37-TiO_2_ NPs, effectively providing the peptide-coated particles with anti-inflammatory properties also without UV illumination. On photocatalytic degradation, most LTA and LPS remain within increasingly densely packed coaggregates, and only a few fragments diffuse into the solution surrounding the lipopolysaccharide-NP coaggregates. Importantly, there seems to be no intermediate degradation, during which lipopolysaccharide fragments are released to an extent, triggering cell activation. In this context and demonstrating the biological importance of such effects, it should be noted that confinement of inflammatory response by coaggregation has previously been observed for the antimicrobial peptide TCP96, which is released from thrombin in response to bacterial protease activity in wound fluids to allow localized inflammation while simultaneously suppressing delocalized inflammatory responses.^66^ Bacterial lipopolysaccharides may also be captured by various other types of nanomaterials, based on either nonspecific binding or binding mediated by moieties specifically recognizing (parts of) such lipopolysaccharides.^67^ Compared to such systems, however, the presently investigated peptide-coated photocatalytic nanoparticles provide the advantage of not only capturing bacterial lipopolysaccharides (in darkness) but also being able to degrade the latter on illumination.

Taken together, the present study shows that peptide-coated TiO_2_ NPs offer interesting opportunities for photocatalytic degradation of bacterial lipopolysaccharides at simultaneously low cell toxicity, with possibilities for confinement of infection and inflammation. Due to limitations in tissue permeation for UV light, areas of particular relevance are those located at the skin or in wounds, as wells as those occurring at mucosal surfaces (e.g., buccal or nasal), that can be conveniently reached by light guides.^68^ Furthermore, the results provide some insight into the mechanism of such photocatalytic degradation, particularly regarding the effects of (i) lipopolysaccharide type, (ii) LPS length, and (iii) the relative importance of polysaccharide and lipid A degradation.

However, numerous issues remain unresolved. For example, whereas similar nanoparticle properties were observed for LL-37 and similar-size polyarginine homopolymer coatings, photocatalytic degradation may be suppressed by overcharging the NP coating (as for polyarginine) (Figure S10), likely through electrostatic arrest, preventing NP insertion into the lipopolysaccharide layers. Further studies are therefore needed to elucidate how peptide properties of importance for lipopolysaccharide interactions of free AMPs translate into the anti-inflammatory effects of the corresponding particle-bound AMPs.

## Conclusions

**Figure 8:**
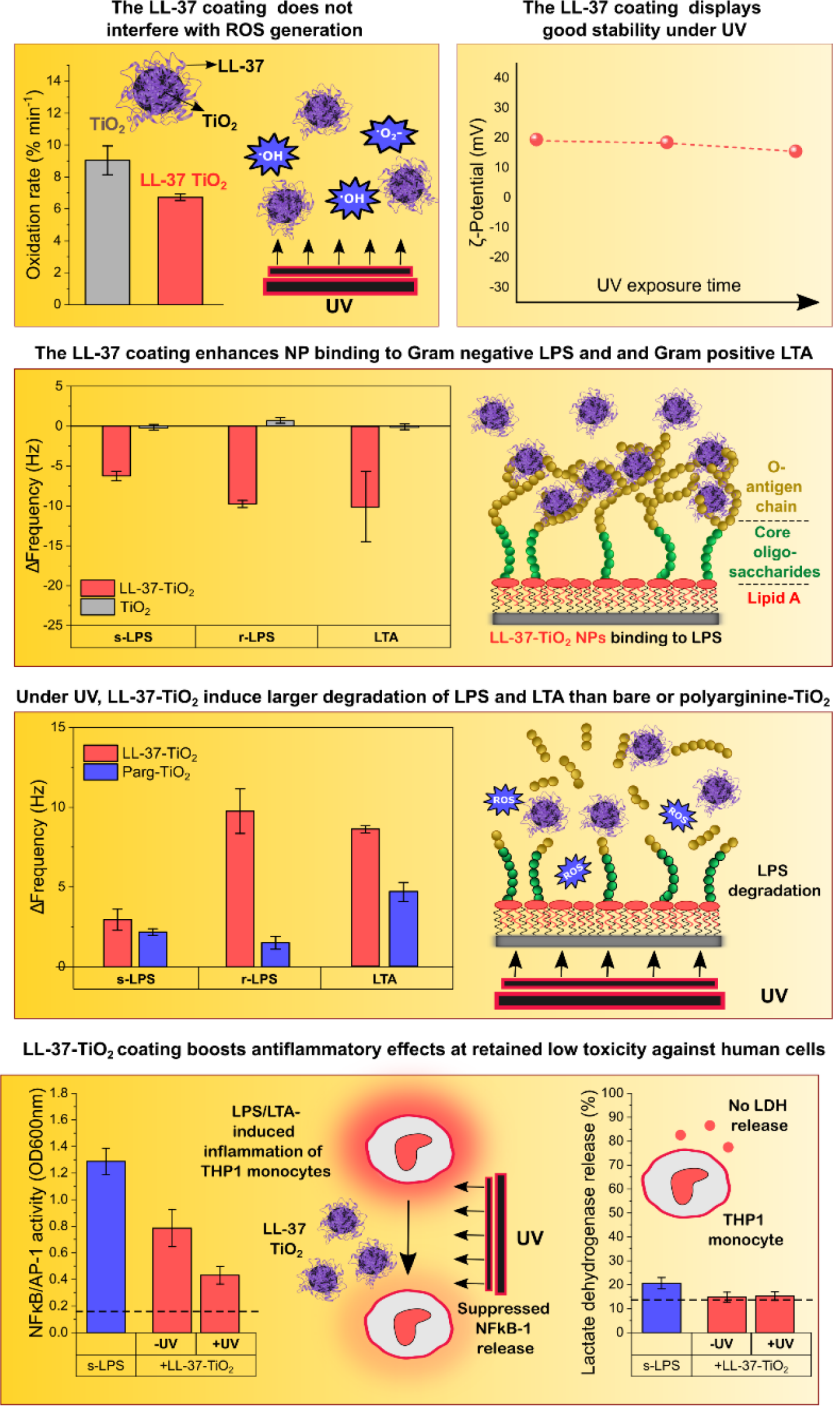
Schematic illustration of key findings of the study: LL-37 coatings on TiO_2_ NPs did not detrimentally interfere with ROS generation and displayed good stability on UV exposure. As a result, binding of net cationic LL-37-TiO_2_ NPs to anionic Gram-negative LPS, its lipid A moiety, and Gram-positive LTA was much higher than that to weakly charged bare TiO_2_ NPs. Mirroring this, LL-37-TiO_2_ displayed potent capturing and UV-induced degradation fragments from LPS and LTA. While qualitatively similar effects were observed for polyarginine-coated NPs, oxidative degradation for all systems was lower than that for LL-37-TiO_2_ NPs, likely due to electrostatic arrest preventing the highly cationic polyarginine NPs from effectively incorporating into the lipopolysaccharide layers. Mirroring the effects observed in model lipopolysaccharide systems, LL-37-coated TiO_2_ NPs displayed boosted anti-inflammatory effects induced by both LPS and LTA at UV illumination, whereas toxicity against human monocytes remained low.

On coating TiO_2_, NPs (IEP ≈ 6.5) were coated with the antimicrobial peptide LL-37 (IEP ≈ 11.2), and the colloidal stability at the physiological pH range was strongly enhanced. Moreover, the peptide coating was stable over at least 2 h of UV illumination and did not detrimentally affect ROS generation. As a result of the positive charge of the peptide-coated NPs, binding to anionic bacterial lipopolysaccharides was strongly promoted. The peptide-coated NPs distributed uniformly over the lipopolysaccharides and resulted in dense coaggregates. On UV-induced degradation, packing constraints induced by chain-like LPS were relaxed, resulting in denser coaggregates as well as in efficient capture of lipopolysaccharide fragments formed. Monitoring structural aspects of these processes, neutron reflectometry showed the LPS degradation of LL-37-TiO_2_ NPs to occur preferentially in its outer O-antigen region. Gram-positive LTA showed qualitatively similar effects as Gram-negative LPS regarding UV-induced degradation, coaggregate densification, and the absence of ordered structure formation. These effects were found to be relevant to the ability of peptide-coated TiO_2_ NPs to suppress inflammatory activation by bacterial lipopolysaccharides. In addition, the peptide-coated TiO_2_ NPs displayed low toxicity against human monocytes, showing the potential of such NPs to effectively localize inflammatory responses at simultaneously low toxicity (Figure 8).

## Supporting Information Available

Calculated SLDs; summaries of structural data obtained from neutron reflectometry experiments; neutron reflectivity curves and best fits; data on oxidation kinetics; and ζ-potential, oxidation assay, synchrotron-SAXS, and cryoTEM results; input parameters used for NR fits and structural parameters obtained from NR fits (PDF)
