# Supplementary material for: Photocatalytic Degradation of Bacterial Lipopolysaccharides by Peptide-Coated TiO2 Nanoparticles
Source: ACS Appl Mater Interfaces. 2024 Oct 23;16(44):60056–69. doi: 10.1021/acsami.4c15706 (PMC11551910; doi:10.1021/acsami.4c15706)
Supplement: Supplementary file 1 — am4c15706_si_001.pdf [file am4c15706_si_001.pdf]

Supporting information

Photocatalytic degradation of bacterial lipopolysaccharides by peptide-coated TiO_2 nanoparticles

Lucrezia Caselli,[1,] Guanqun Du[1], Samantha Micciulla[2], Tanja Traini[3], Federica Sebastiani[1,3], Ragna Guldsmed Diedrichsen[3], Sebastian Köhler[4], Maximilian W. A. Skoda[5], Mariena van der Plas[3], and Martin Malmsten[1,3]*

[1]Department of Physical Chemistry 1, Lund University, SE-22100 Lund, Sweden (email:caselli@csgi.unifi.it)

[2]Institut Laue–Langevin, CS 20156, 38042 Grenoble Cedex 9, France

[3]Department of Pharmacy, University of Copenhagen, DK-2100 Copenhagen, Denmark

[4]LINXS Institute of Advanced Neutron and X-ray Science, Scheelevagen 19, 22370 Lund, Sweden

[5] ISIS Pulsed Neutron and Muon Source, Rutherford Appleton Laboratory, OX11 0QX, Harwell, UK

[*]Corresponding author

KEYWORDS: Antimicrobial peptide, lipopolysaccharide, photocatalysis, oxidation, TiO_2

Scheme S1 Schematic illustration of the model applied to fit the NR data. For smooth LPS adsorbed on pre-coated SiO_2-OTS surfaces (in the absence as well as in the presence of NPs), a 3-layer model was employed. This comprises a single and fully interdigitated OTS/lipid A hydrophobic layer on top of the SiO_2 surface, followed by a layer of core oligosaccharides, and an outer layer of O-antigen chains.

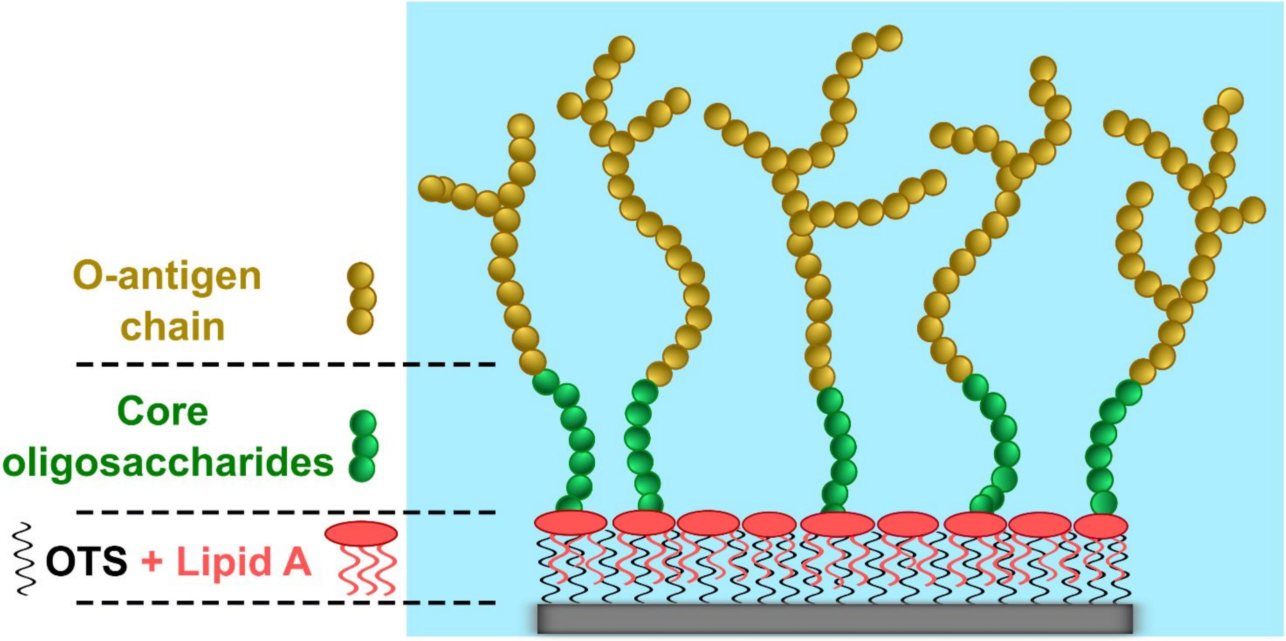

Table S1. Summary of structural data obtained from the NR fits for the hydrophobic OTS layer, pre-formed onto SiO_2 blocks. The value of the SLD of the layer is shown in bold and was assumed constant and taken from literature (1). Shown also are fitted structural parameters, including thickness (Thick), hydration (Hyd) and roughness (Rough) of the layer. These parameters were obtained fitting the NR profiles by using the Genetic Optimization method the available on the Motofit analysis package within the software IGOR Pro (2,3), according to a single layer model accounting for the OTS layer covalently bound to the SiO_2 surface. A Monte Carlo error analysis allowing for refitting data 200 times was employed to minimize the uncertainty associated to data fitting (4).

SLD (10^{-6} Å^{-2})	Thick (Å)	Hyd (vol%)	Rough (Å)
**-0.35**	30.9 ± 0.1	0.8 ± 0.2	5.9 ± 0.1

Table S2. Summary of structural data obtained from the NR fits for supported smooth LPS before and after **bare TiO_2** NP deposition, as well as after 2 h of UV exposure. Input parameters (i.e., SLD values for the different layers) are shown in bold, assumed constant with fixed values taken from previous studies (1,5). For core oligosaccharide and O-antigen chain layers, the variation in the SLD in the different contrasts (d-, qm- and h-buffer, i.e., 10 mM Tris buffer in 100% v/v D_2O, 68.6/31.4% v/v D_2O/H_2O, and 100% v/v H_2O, respectively) due to partial hydrogen exchange was considered according to previous studies (1,5). Additionally, the SLD of the mixed OTS/Lipid A layer was taken as the arithmetic mean of OTS and lipid A individual components. Shown also are calculated structural parameters, including thickness (Thick), hydration (Hyd, referring to volume percentage), roughness (Rough) for each layer. These parameters were obtained fitting the NR profiles by using the Genetic Optimization method the available on the Motofit analysis package within the software IGOR Pro (2,3), according to a 3-layers model (**Scheme S1**), which accounts for: (1) a fully interdigitated OTS/Lipid A layer, (2) a core oligosaccharide layer, and (3) an outer layer of O-antigen chains. A Monte Carlo error analysis allowing for refitting data 200 times was employed to minimize the uncertainty associated to data fitting (4). Data taken from (6).

	SLD (10^{-6} Å^{-2})	Thick (Å)	Hyd (vol %)	Rough (Å)
**OTS + Lipid A**				
Initial	**-0.37**	32.8 ± 0.2	0.06 ± 0.05	4.9 ± 0.2
TiO_2	(OTS=**-0.35**	29.2 ± 0.2	0.4 ± 0.2	6.4 ± 0.3
+2h UV	Lipid A=**--0.4)**	30.1 ± 0.1	0.4 ± 0.2	5.4 ± 0.3
**Core oligosaccharides**				
Initial	**1.85** (h)	10.2 ± 0.2	47 ± 3	4.1 ± 0.1
TiO_2	**3.79** (qm)	14 ± 2	47 ± 7	4.6 ± 0.3
+2h UV	**4.70** (d)	13 ± 1	45 ± 6	5.3 ± 0.4
**O-antigen chain**				
Initial	**1.57** (h)	103 ± 9	97 ± 1	5.3 ± 0.2
TiO_2	**3.19** (qm)	106 ± 12	95 ± 3	8 ± 1
+2h UV	**3.94** (d)	84 ±17	96 ± 1	8 ± 1

Table S3. Summary of structural data obtained from the NR fits for supported smooth LPS before and after **LL-37-TiO$_2$** NP deposition, as well as after 2 h of UV exposure. Input parameters (i.e., SLD values for the different layers) are shown in bold, assumed constant with fixed values taken from previous studies (1,5). For core oligosaccharide and O-antigen chain layers, the variation in the SLD in the different contrasts (d-, qm- and h-buffer, i.e., 10 mM Tris buffer in 100% v/v D_2O, 68.6/31.4% v/v D_2O/H_2O, and 100% v/v H_2O, respectively) due to partial hydrogen exchange was considered according to previous studies (1,5). Additionally, the SLD of the mixed OTS/Lipid A layer has been taken as the arithmetic mean of OTS and lipid A individual components. Shown also are calculated structural parameters, including thickness (Thick), hydration (Hyd, referring to volume percentage), roughness (Rough) for each layer. These parameters were obtained fitting the NR profiles by using the Genetic Optimization method the available on the Motofit analysis package within the software IGOR Pro (2,3), according to a 3-layers model (**Scheme S1**), which accounts for: (1) a fully interdigitated OTS/Lipid A layer, (2) a core oligosaccharide layer, and (3) an outer layer of O-antigen chains. A Monte Carlo error analysis allowing for refitting data 200 times was employed to minimize the uncertainty associated to data fitting (4).

	SLD (10^{-6} Å^{-2})	Thick (Å)	Hyd (vol %)	Rough (Å)
**OTS + Lipid A**				
Initial	**-0.37**<br>(OTS=**-0.35**<br>Lipid A=**--0.4)**	27.8 ± 0.1	0.5 ± 0.3	6.5 ± 0.2
LL-37-TiO$_2$		28.3 ± 0.1	0.3 ± 0.2	7.3 ± 0.1
+2h UV		27.8 ± 0.2	0.6 ± 0.2	5.1 ± 0.1
**Core oligosaccharides**				
Initial	**1.85** (h)<br>**3.79** (qm)<br>**4.70** (d)	16.3 ± 0.8	34 ± 3	5 ± 0.3
LL-37-TiO$_2$		14.2 ± 05	32 ± 2	4.2 ± 0.1
+2h UV		10 ± 1	36 ± 5	6.7 ± 0.2
**O-antigen chain**				
Initial	**1.57** (h)<br>**3.19** (qm)<br>**3.94** (d)	124 ± 24	97 ± 1	9 ± 1
LL-37-TiO$_2$		40 ± 7	94.3 ± 0.7	5.7 ± 0.4
+2h UV		9 ± 1	59 ± 8	10 ± 1

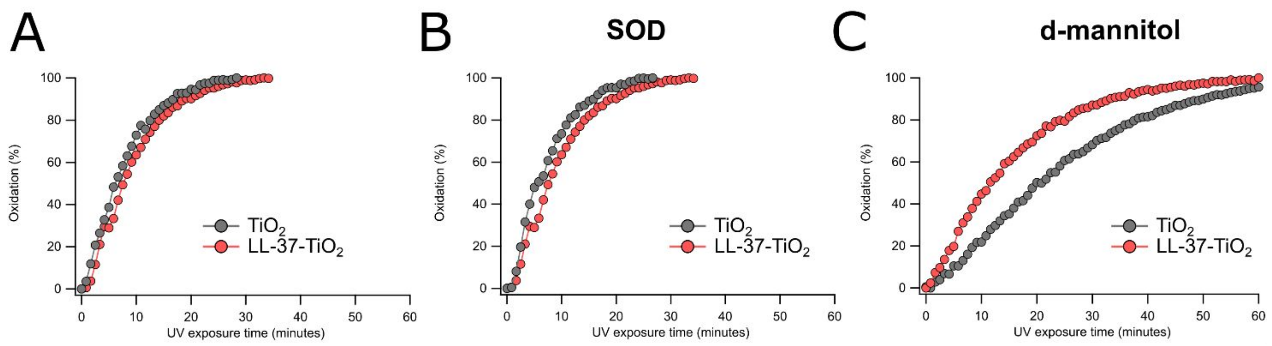

Figure S1. Representative C_{11}-BODIPY oxidation kinetics, showing effects of 100 ppm bare TiO_2 NPs (grey) and LL-37-TiO_2 NPs (red) on +PG LUVs subjected to *in situ* UV exposure in the absence (A), and presence of SOD (•O_2^- scavenger, +50 U/mL) (B), ord-mannitol (•OH scavenger, 500 mM) (C) in 10 mM Tris, pH 7.4. (n=3).

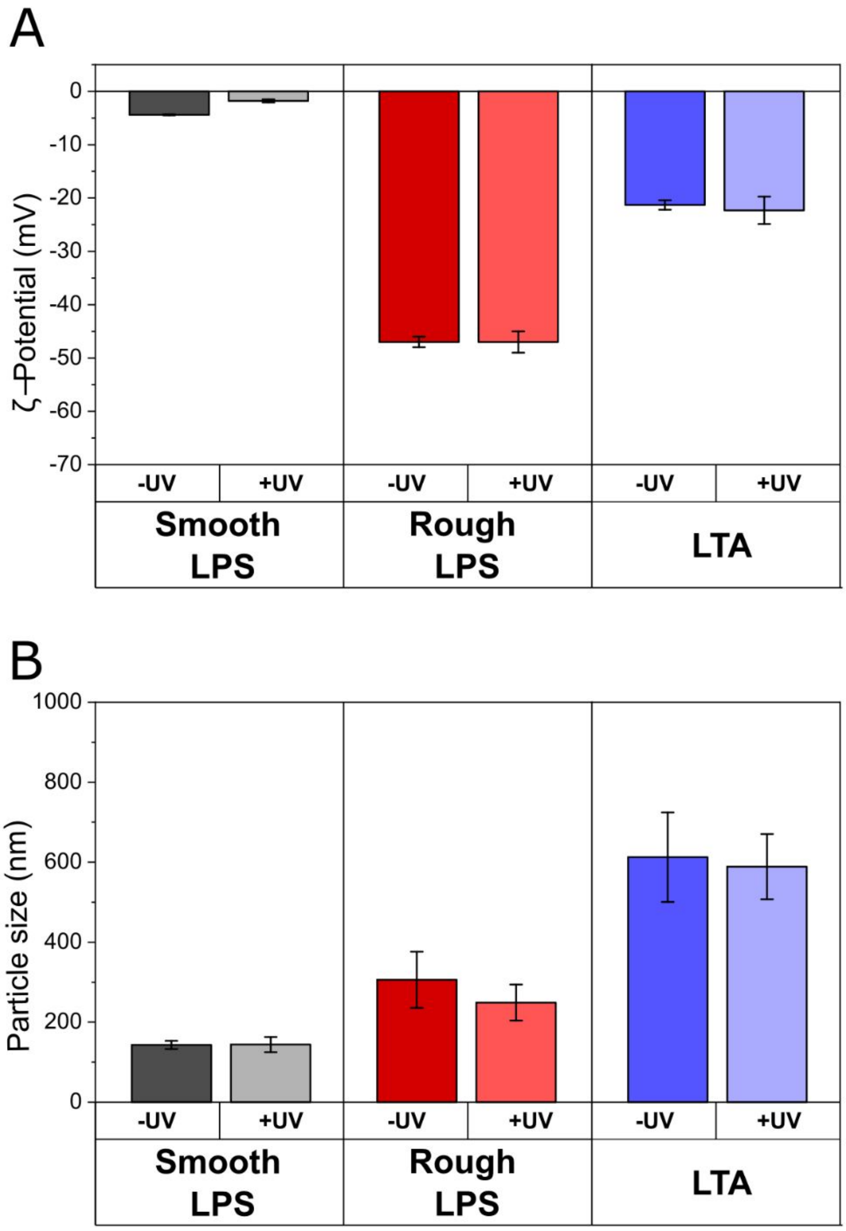

Figure S2. ζ-potential (A) and average particle size (B) for 100 ppm smooth LPS (left), rough LPS (middle), or LTA (right) in the absence of bare TiO_2 NPs, before and after 2 h of UV illumination in 10 mM Tris, pH 7.4. (n=3).

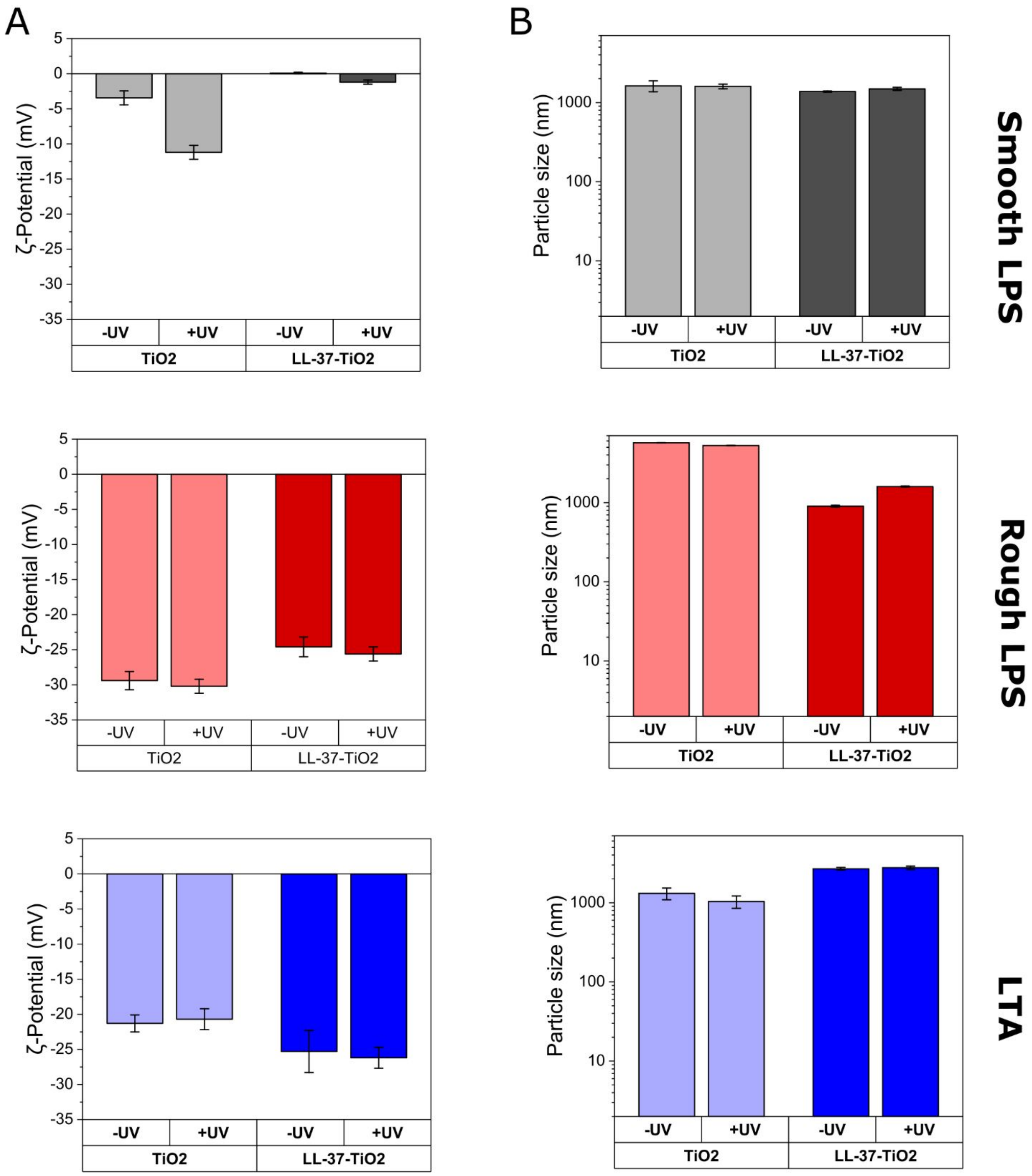

Figure S3. ζ-potential (A) and average particle size (B) obtained on mixing 100 ppm bare or LL-37-TiO_2 NPs with 100 ppm smooth LPS (top), rough LPS (middle), or LTA (bottom), as well as effects of UV illumination for 2 h in 10 mM Tris, pH 7.4. (n=3)

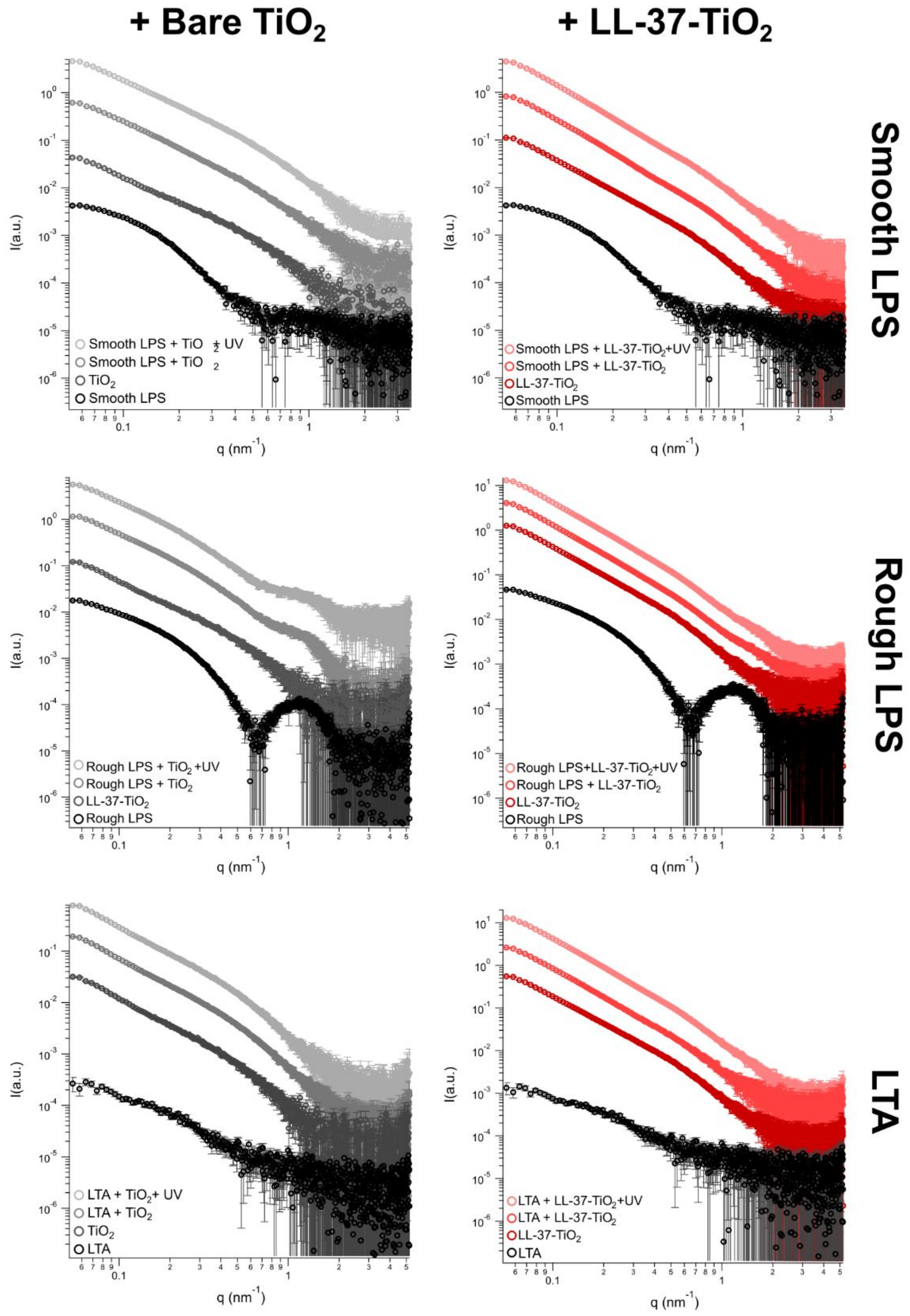

Figure S4. SAXS profiles of 100 ppm smooth LPS (top), rough LPS (middle) and LTA (bottom) incubated for 2 h with either bare (left) or LL-37-coated TiO_2 NPs (100 ppm) in the absence or presence of UV illumination, in 10 mM Tris, pH 7.4. SAXS profiles of bare LPS and LTA, as well as bare or LL-37-coated TiO_2 NPs in the same buffer, are also included as control samples. Measurements were performed at 25°C.

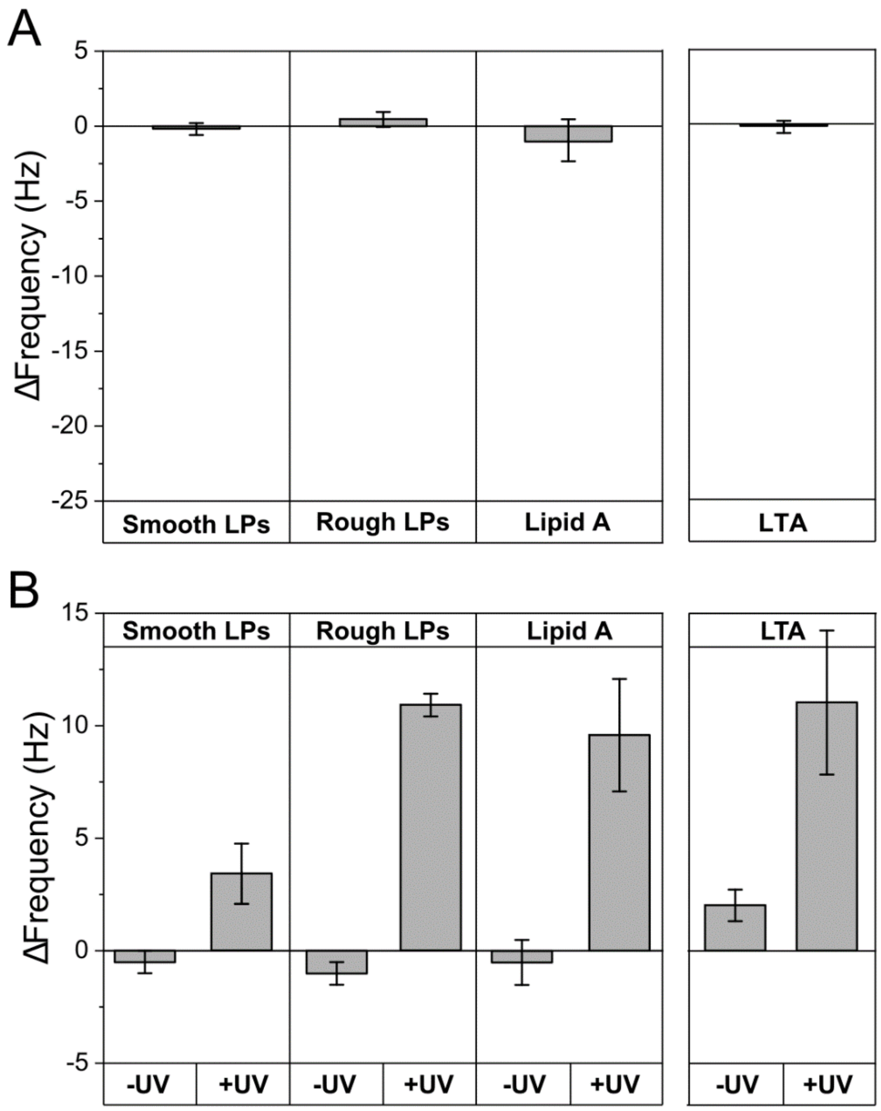

Figure S5. QCM-d results on (A) adsorption of 100 ppm of bare TiO_2 NPs to hydrophobic polystyrene surfaces coated with smooth LPS, rough LPS, lipid A and LTA (prepared as in **Figure 4**). Shown in (B) are results on the subsequent oxidative degradation of such layer after 2 hours of UV illumination. ΔF=0 corresponds to the Frequency shift for the smooth LPS, rough LPS and Lipid A layers right before NP binding (A) or UV illumination (B). Measurements were performed in 10 mM Tris, 150 mM NaCl, pH 7.4 (n=3).

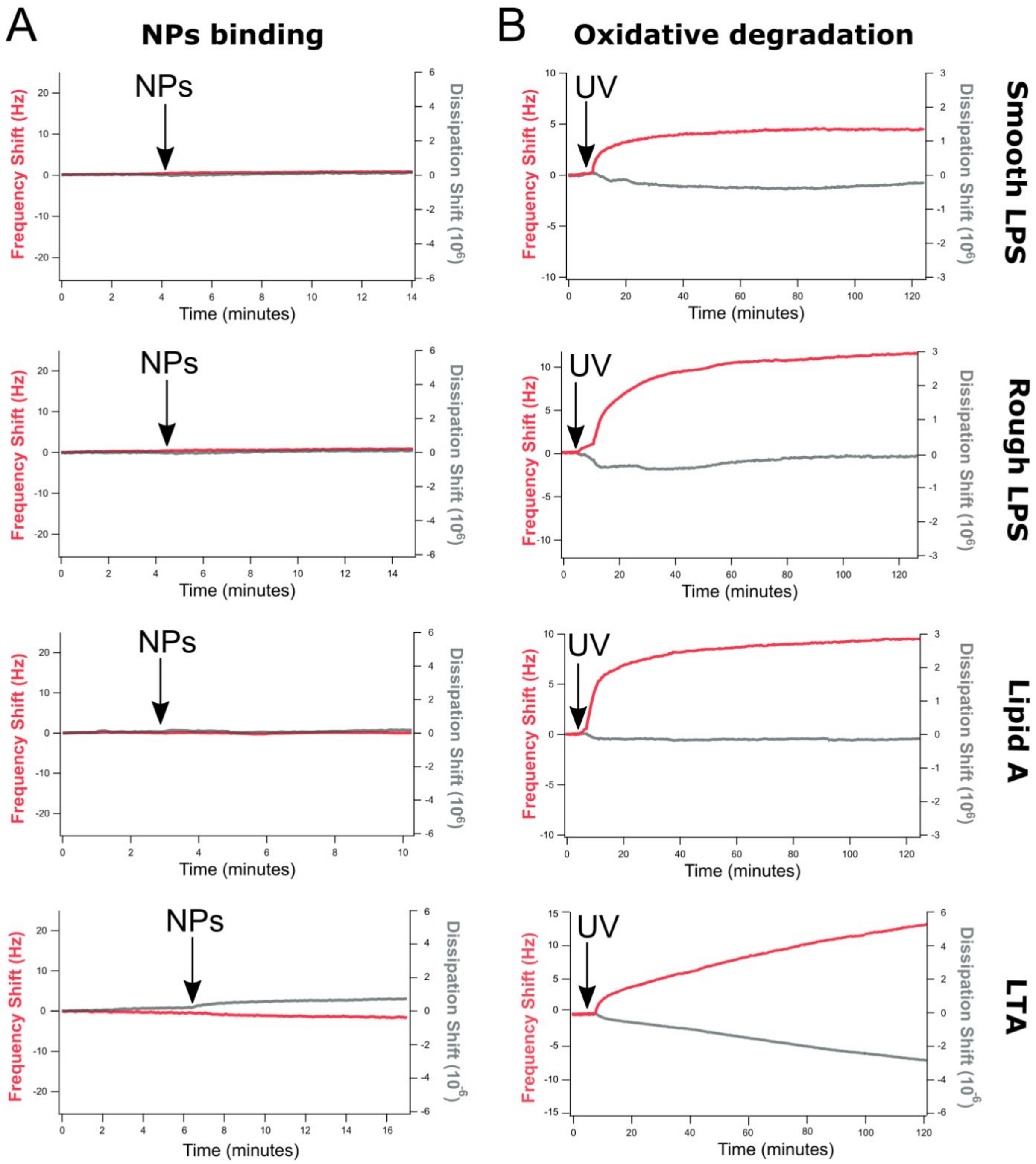

Figure S6. Representative QCM-d profiles showing the 7th overtone of ΔFrequency (Hz) and ΔDissipation ($\cdot 10^{-6}$) for (A) 100 ppm **bare TiO_2** NPs binding to smooth LPS, rough LPS, lipid A, and LTA adsorbed on polystyrene and (B) subsequent oxidative degradation induced upon UV illumination applied for 2 hours (right). ΔF=0 and ΔD=0 correspond to Frequency and Dissipation shifts for smooth LPS, rough LPS, lipid A and LTA layers right before NP binding (A) or UV illumination (B). Measurements were performed in 10 mM Tris, 150 mM NaCl, pH 7.4 (n=3).

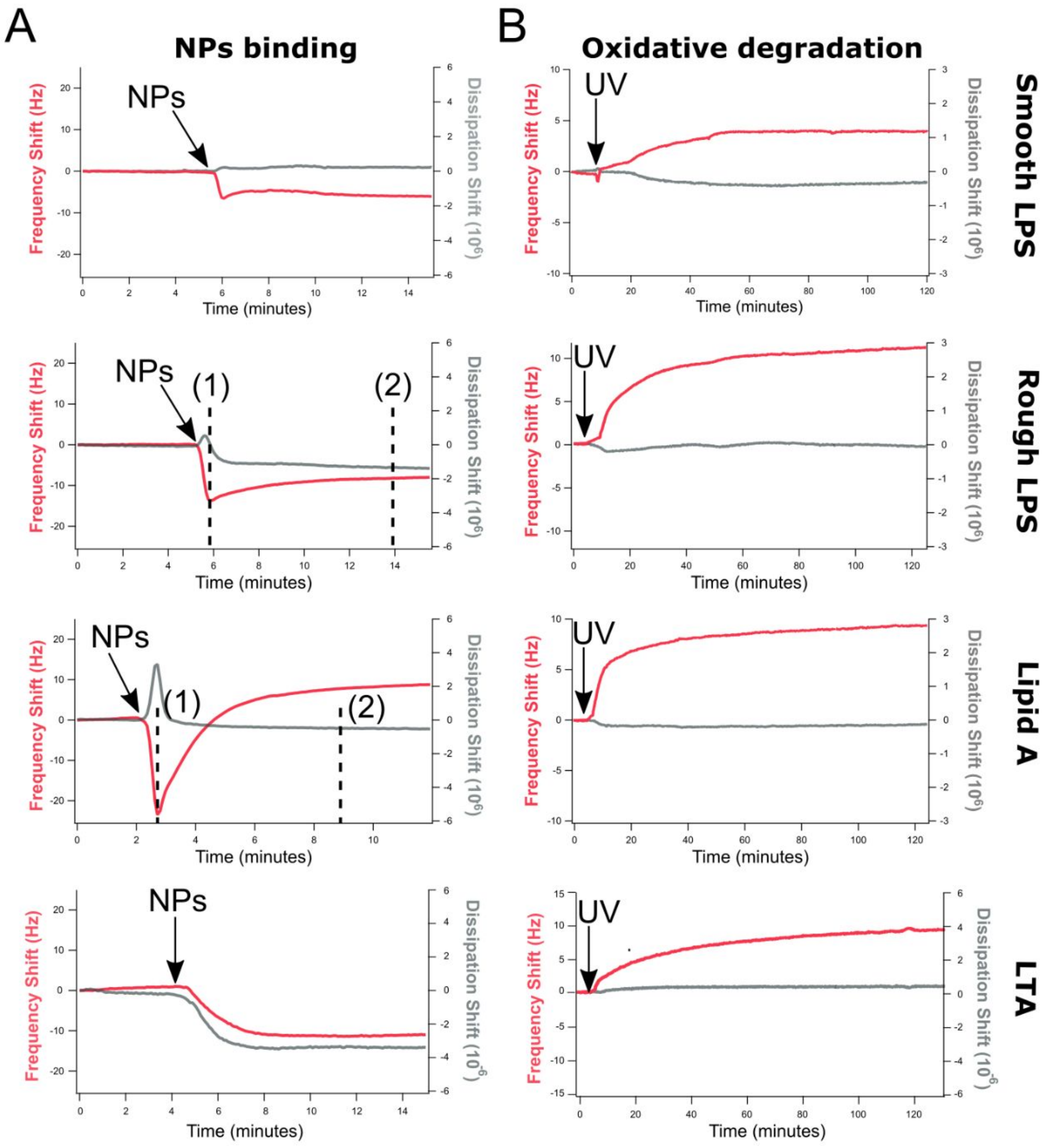

Figure S7. Representative QCM-d ΔFrequency (Hz) and ΔDissipation ($\cdot 10^{-6}$) profiles showing (A) 100 ppm **LL-37-TiO$_2$** NPs binding to smooth LPS, rough LPS, lipid A, and LTA adsorbed on polystyrene and (B) subsequent oxidative degradation induced upon UV illumination applied for 2 hours (right). ΔF=0 and ΔD=0 correspond to Frequency and Dissipation shifts for smooth LPS, rough LPS, lipid A and LTA layers right before NP binding (A) or UV illumination (B). Measurements were performed in 10 mM Tris, 150 mM NaCl, pH 7.4 (n=3). When LL-37-coated TiO_2 NPs are injected into the measurement chamber (left), a minimum in ΔF is reached soon after NP injection ((1)), indicating a maximum in NPs adsorption for rough LPS, and lipid A; this is followed by a slower increase in ΔF, consistent with a partial removal of material, which stops within 10 minutes of incubation ((2)).

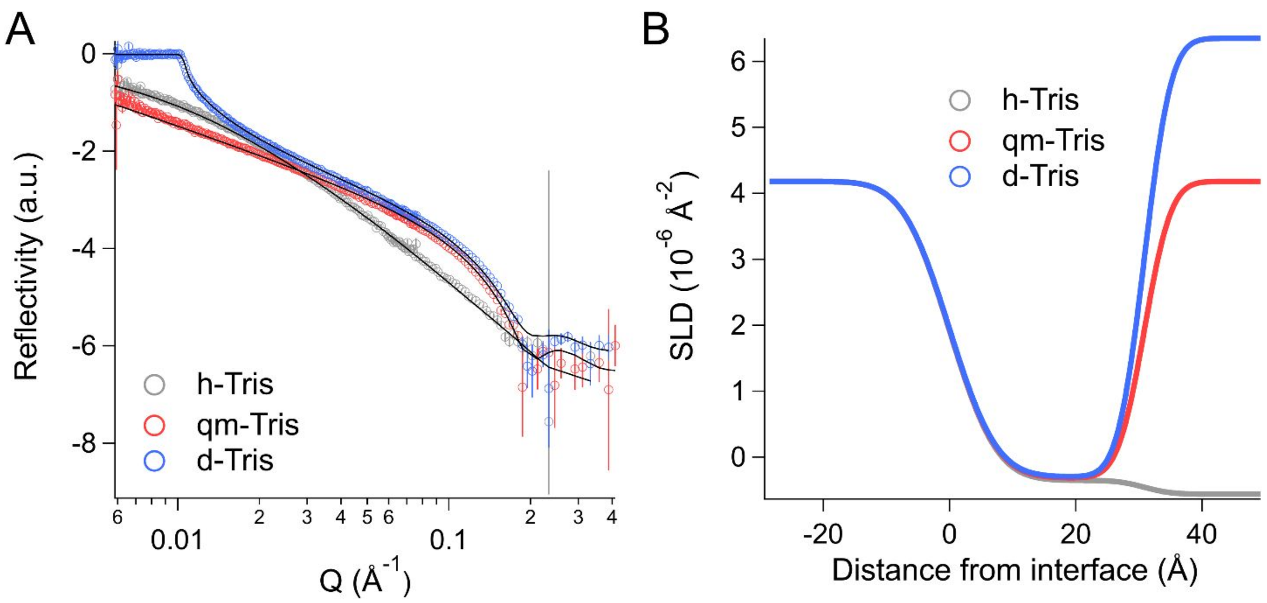

Figure S8. Neutron reflectivity curves with best model fits (A) and corresponding SLD profiles (B) for a supported OTS layer before LPS deposition in 10 mM d-Tris, qm-Tris, and h-Tris, pH 7.4. Adapted from (6).

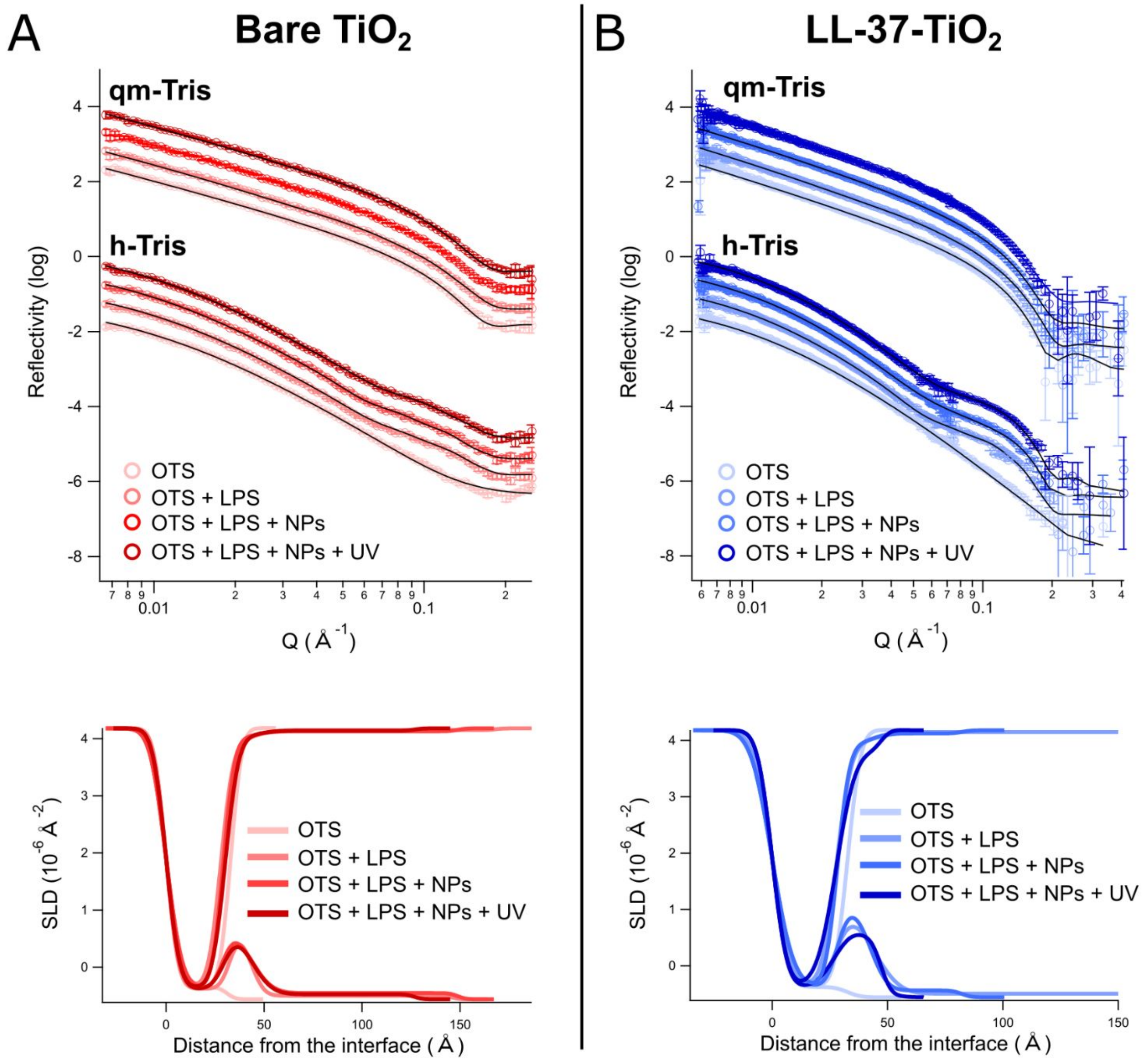

Figure S9. Neutron reflectivity curves with best model fits (upper) and corresponding SLD profiles (lower) for smooth LPS layers before and after incubation with bare TiO_2 NPs (A) and LL-37-TiO_2 NPs (B). Shown are also reflectivity curves with best model fits and SLD profiles for the corresponding systems after 2 h of *in situ* UV exposure. All experiments were performed in 10 mM Tris buffer, pH 7.4, at a nanoparticle concentration of 100 ppm. Curves are shown for two different buffer contrasts, h-tris and qm-Tris, and data for the latter are offset by $3 \cdot 10^{-1}$ for clarity. The grey box in the SLD profiles indicates the position of the silicon block and reflecting interface, consisting of bulk Si and a SiO_2 layer. Data for bare TiO_2 NPs (A panel) are adapted from (6).

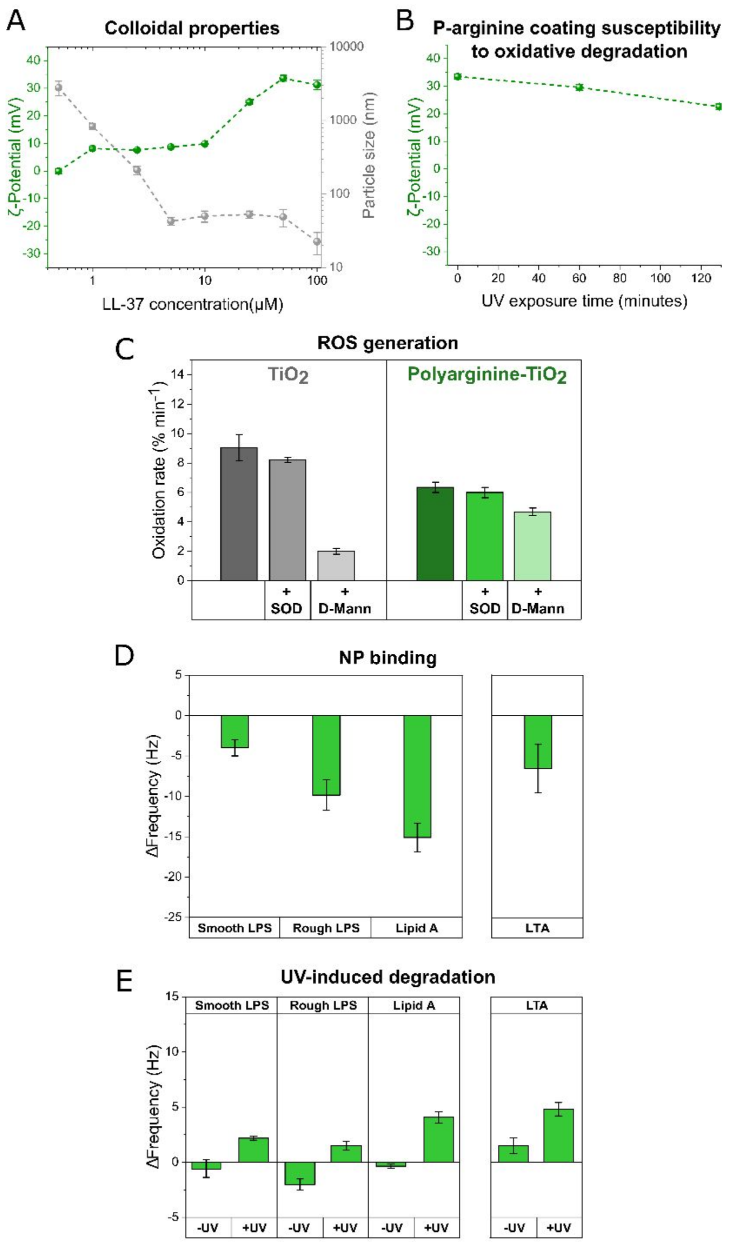

Figure S10. Concept generalization. (A) On coating TiO_2 NPs by highly positively charged polyarginine at pH 7.4, colloidal stability is strongly improved. (B) The peptide coating displays good integrity on UV exposure for 1-2 h (as inferred from the ζ-potential of polyarginine-TiO_2 NPs) and does not detrimentally interfere with ROS generation (C). As a result, binding of polyarginine-TiO_2 NPs to smooth LPS, rough LPS, lipid A, and LTA was strongly promoted (D). Oxidative degradation under UV exposure was comparable for smooth LPS, rough LPS, lipid A, and LTA, but smaller throughout for polyarginine-TiO_2 NPs (E) than for LL-37-TiO_2 NPs (**Figure 5B**).

References

1. Rodriguez-Loureiro, I., Latza, V.M., Fragneto, G. and Emanuel Schneck and and Schneck, E. Conformation of Single and Interacting Lipopolysaccharide surfaces bearing O-side chains. Biophys J. 2018, 116, 114, 1624-1635.

2. Nelson, A. Motofit – integrating neutron reflectometry acquisition, reduction and analysis into one, easy to use, package. J. Phys. Conf. Ser. 2010, 251, 012094.

3. Nelson, A. Co-refinement of multiple contrast neutron / X-ray reflectivity data using MOTOFIT. J. Appl. Cryst. 2006, 39, 273-276.

4. Heinrich, F., Ng, T., Vanderah, D.J., Shekar, P., Mihailescu, M., Nanda, H., and Lösche, M. A new lipid anchor for sparsely tethered bilayer lipid membranes. Langmuir 2009, 25, 4219-4229.

5. Micciulla, S., Gerelli, Y., and and Schneck, E. Structure and conformation of wild-type bacterial lipopolysaccharide layers at air-water interfaces. Biophys J. 2019, 116, 1259–1269.

6. Caselli, L., Traini, T., Micciulla, S., Sebastiani, F., Köhler, S., Nielsen, E. M., Guldsmed Diedrichsen, R., Skoda, M.W. A., Malmsten, M. Antimicrobial Peptide Coating of TiO_2 Nanoparticles for Boosted Antimicrobial Effects. Advanced Functional Materials 2024, 2405047.
